# Surface functionalisation significantly changes the physical and electronic properties of carbon nano-dots†
†Electronic supplementary information (ESI) available. See DOI: 10.1039/c8nr03430c
‡
‡The raw data supporting this article are archived in the University of Bristol's Research Data Storage Facility (DOI: 10.5523/bris.30s3i070zb0qv2tipisop5a1h4)


**DOI:** 10.1039/c8nr03430c

**Published:** 2018-07-10

**Authors:** Thomas A. Swift, Marta Duchi, Stephen A. Hill, David Benito-Alifonso, Robert L. Harniman, Sadiyah Sheikh, Sean A. Davis, Annela M. Seddon, Heather M. Whitney, M. Carmen Galan, Thomas A. A. Oliver

**Affiliations:** a School of Chemistry , Cantock's Close , University of Bristol , BS8 1TS , UK . Email: tom.oliver@bristol.ac.uk ; Email: m.c.galan@bristol.ac.uk; b School of Biological Sciences , Life Sciences Building , Tyndall Avenue , University of Bristol , BS8 1TH , UK; c Bristol Centre for Functional Nanomaterials , HH Wills Physics Laboratory , Tyndall Avenue , University of Bristol , BS8 1TL , UK; d School of Physics , HH Wills Physics Laboratory , Tyndall Avenue , University of Bristol , BS8 1TL , UK

## Abstract

Biomolecule functionalisation of carbon nano-dots (CDs) greatly enhances their biocompatibility and applicability, however, little is known about their molecular structure. Using an arsenal of spectroscopic and analytical techniques, we provide new insights into the physical and electronic structure of uncoated and glycan-functionalised CDs. Our studies reveal that surface functionalisation does not always result in a homogenous corona surrounding the core, and the choice of carbohydrate significantly affects the electronic structure of the surface CD states. Further, the average surface coverage of an ensemble of CDs can be probed *via* transient absorption spectroscopy. These findings have implications for CDs targeted at interactions with biological systems or local sensors.

## Abstract 2

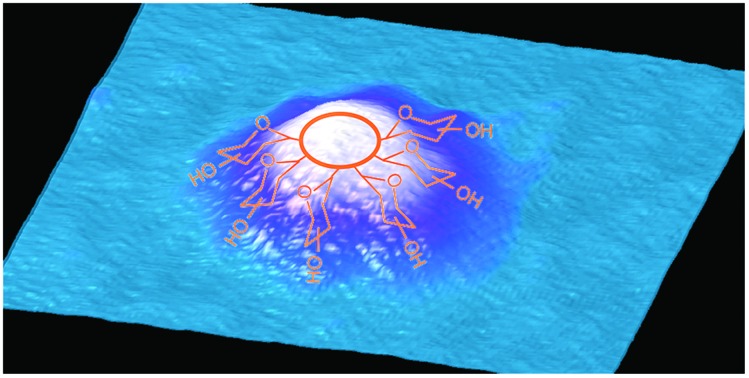
The electronic structure of glycan functionalised carbon nano-dots is greatly affected by the choice of carbohydrate.

## 

One of the main targets within nanoscience is the development of fluorescent, non-toxic, nanomaterials that can easily be synthesised and selectively functionalised. Since the serendipitous discovery of carbon nano-dots (CDs) in 2004, significant advances in the synthesis and functionalisation of CDs have seen these non-toxic nanomaterials surpass traditional cadmium based quantum dots (QDs) as fluorescent platforms for a number of biological and chemical studies,^1^–^8^ including many interesting applications that range from sensing of materials,^9^–^14^ to bacteria and live cell imaging, fluorescent labeling,^2^,^15^,^16^ targeting cancer cells^17^ and gene delivery.^18^ Furthermore, their photo-stability and broad absorption throughout the visible and ultraviolet has enabled their use as light harvesting and photo-protective elements that enhance the efficiency of perovskite solar cells,^19^ optoelectronics,^20^ and photocatalysts.^7^

Water-soluble CDs with high fluorescence quantum yields have been produced from a range of organic sources including carbohydrates using an array of methods such as thermal decomposition, chemical oxidation, hydrothermal oxidation under autoclave and microwave-assisted conditions.^1^,^3^,^21^–^24^ Carbohydrate functionalised nanoparticles have been used by our group and others to probe and enable interactions with biological systems and enhance cell internalisation of nanoparticles.^25^–^29^ Furthermore, our group recently reported the three minute microwave-assisted synthesis of non-toxic water-soluble CDs from a carbohydrate starting material, providing a low-cost and practical synthetic route to amine-coated CDs ready for biomolecule conjugation.^30^ Transmission electron microscopy (TEM) and Raman spectroscopic analysis of these CDs demonstrated that the core is comprised of a crystalline sp^3^-carbon domain decorated by layer(s) of poly-aromatic moieties.^30^ We also showed that lactose-functionalised CDs could be used as intracellular non-toxic fluorescent labels.

Despite a number of studies using CDs, only a few have examined the connection between the physical structure of CDs and their fluorescent properties.^7^,^31^–^34^ Previous work investigating the electronic structure and photoluminescence mechanisms of CDs has shown that most CDs do not behave like traditional QDs, but instead should be considered as nanoscale assemblies of fluorophores,^32^,^35^ with associated large Stokes shifts.^31^,^33^,^36^–^38^ The fluorescent properties of the CDs were found to be dependent on the surface structure, which likely varies depending on the chosen synthetic route^39^ and thus can be tuned by the formation of CD microstructures.^40^ The prevailing consensus has been that functionalisation of CDs results in a homogeneously coated surface, and that conjugation with biomolecules does not affect or change the electronic structure of CDs, *i.e.* absorption and fluorescence.^18^,^41^,^42^ However, if the surface functionalisation is inhomogeneous, three-dimensional biomolecules will bind in different orientations and with different affinities, which consequently will alter any modulation of biological function.

Herein, we report the synthesis of a series of biologically-relevant glycan-coated CDs and the effect of functionalisation on the molecular structure of CDs, *via* a range of analytical and spectroscopic techniques.

Glycan-coated CDs **5a–e** were prepared following a modified version of our previously reported synthesis,^30^ as illustrated in Scheme 1. Further details are given in the Experimental procedures section and ESI.† Microwave-assisted reaction of 4,7,10-trioxa-1,13-tridecanediamine (TTDDA) and glucosamine hydrochloride **1** in water yielded amine-functionalised CDs **2**, which were then carboxylic acid-functionalised *via* the ring-opening of succinic anhydride prior to 1,1′-carbonyldiimidazole (CDI)-mediated amide coupling with 1-amino glycosides **4a–e**. These specific glycosides were chosen for their biological relevance and applications and to explore the effect of different hydrophobic and hydrophilic regions formed by the different orientation of hydroxyl functional groups (axial *vs.* equatorial and/or mono- *vs.* disaccharide). Other carbohydrates such as trisaccharides (*e.g.* maltotriose) or polysaccharides (*e.g.* cellulose) were not used to functionalise the CDs due to their aggregation in water and their poor solubility in less polar solvents.

**Scheme 1:**
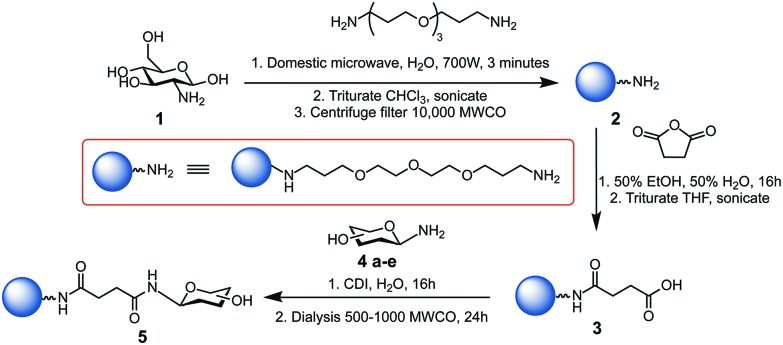
Synthesis of glycan-functionalised CDs **5a–e** using 1-amino-glycosides: glucose **4a**, mannose **4b**, galactose **4c**, maltose **4d**, and lactose **4e** scaffolds.^28^

^1^H NMR, ^1^H–^13^C HSQC, diffusion ordered spectroscopy (DOSY) (Fig. S1–S12†), Fourier-transform infrared spectroscopy (Fig. S13–S18†) of the resulting functionalised CDs, confirmed that the structure of the glycosides remained intact upon conjugation to CDs. It is important to note that a single batch of acid-functionalised CDs was used to conjugate with an excess of the different 1-aminoglycosides. Glycan-functionalisation was verified by a phenol-sulphuric acid assay (see Table S2†), and small differences were observed – the homogeneity of the glycan corona may still be attributed to differences in glycan structure.

A range of analytical techniques were used to characterise the CD particle size distributions and surface structure. Transmission electron microscopy (TEM) measurements of 500 naked carbon nano-dots returned a core radius of 1.3 ± 0.2 nm (error quoted as standard deviation, see histogram in Fig. S19†). TEM images revealed lattices within the CDs, with spacings consistent with sp^3^ crystalline carbon (Fig. S26 and Table S3†). For the glycan functionalised CDs, TEM imaging returned slightly increased radii for the nano-particles (Fig. S20–S31†), and in accord with our previous work.^30^ Additional measurements using small-angle X-ray scattering (SAXS) returned a core radius of 2.8 ± 0.2 nm (errors quoted from the 90% confidence interval returned by Guinier analysis fitting, see Fig. S32†). The larger radius extracted from SAXS measurements is because the measurement is sensitive to both aromatic (surface) and crystalline (core) structures of CD nanoparticles, unlike TEM which is only capable of probing the latter. Atomic-force microscopy (AFM) measurements of the core returned a radii of 3 ± 1 nm (762 particles measured, error quoted as standard deviation, Fig. S34 and S35†).^43^ Together this information provides us with a clearer physical structure for the naked CDs: a ∼1.5 nm crystalline sp^3^ domain surrounded by a ∼1.4 nm aromatic shell. Importantly, neither TEM, or SAXS are sufficiently sensitive to characterise the functionalised glycan-corona, due to the low electron densities associated with the surface carbohydrates.

AFM studies were therefore critical in imaging and determining the homogeneity of the CD surface functionalisation. Fig. 1(A) displays an AFM image of the naked CD core, which forms the scaffold for all subsequent carbohydrate functionalisation. For the functionalised dots, a core region and a functionalisation area extending from the core was observed, as exemplified by height profiles extracted from AFM data (Fig. S35–S43†). The AFM images reveal, however, varying morphology of the glycan-corona on the CD surface: the corona surrounding the core of glucose **5a**, mannose **5b** and lactose **5e** CDs (Fig. 1B, C and F) is fairly uniform, whereas galactose **5c** and maltose **5d** functionalisation resulted in inhomogeneous surface coverage. It is critical to note that these are AFM images of individual CDs, and additional images of each CD species (Fig. S35–S40†) emphasise the inhomogeneous surface coating of CDs. AFM mapping of the tip–sample adhesion interaction (Fig. S36–S41†) assisted in the assignment of specific regions of the CDs into crystalline cores, associated with low-adhesion, and far more adhesive surface functionalised regions. Again, like the topographical AFM images, these data demonstrate that the surface functionalisation varies immensely between individual CDs.

**Fig. 1:**
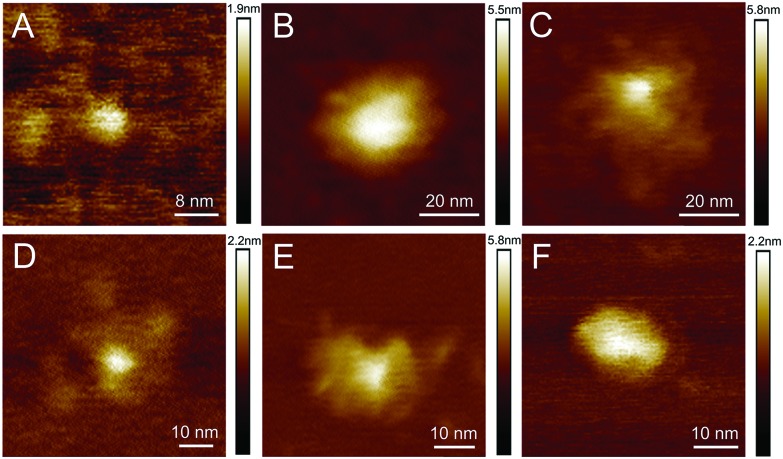
Representative topographic AFM images of CDs with different functionalisations: (A) core, (B) glucose, (C) mannose, (D) galactose, (E) maltose, (F) lactose. Additional images are given in Fig. S35–S40.†

The electronic structure of the naked and functionalised CDs was investigated using both steady state absorption and fluorescence spectroscopy (Fig. S44 and S45†). These spectra are similar to those disseminated by other groups using microwave syntheses with C, N and O containing feed stocks.^31^,^32^,^43^

Functionalisation of the CDs with the five different glycan groups did not affect the strong absorption bands centred at 273 nm and ∼200 nm (see Fig. S44†). However, the longer-wavelength part of the absorption spectra is far more sensitive to the carbohydrate functionalisation. The implications are two-fold: (i) the *λ* > 350 nm region of the absorption spectrum must correspond to excitation located in aromatic surface domains of the CDs as this part of the spectrum is altered upon changing the glycan functionalisation, and (ii) that carbohydrate functionalisation can affect the physical/electronic structure of the surface, rather than acting merely as passivating ligands, as per traditional QDs.^44^ Excitation–emission fluorescence correlation spectra corroborate this view (Fig. S45†). The fluorescence maxima lies at ∼465 nm (*e.g.* mannose in Fig. 2) for excitation at *λ* > 365 nm.

**Fig. 2:**
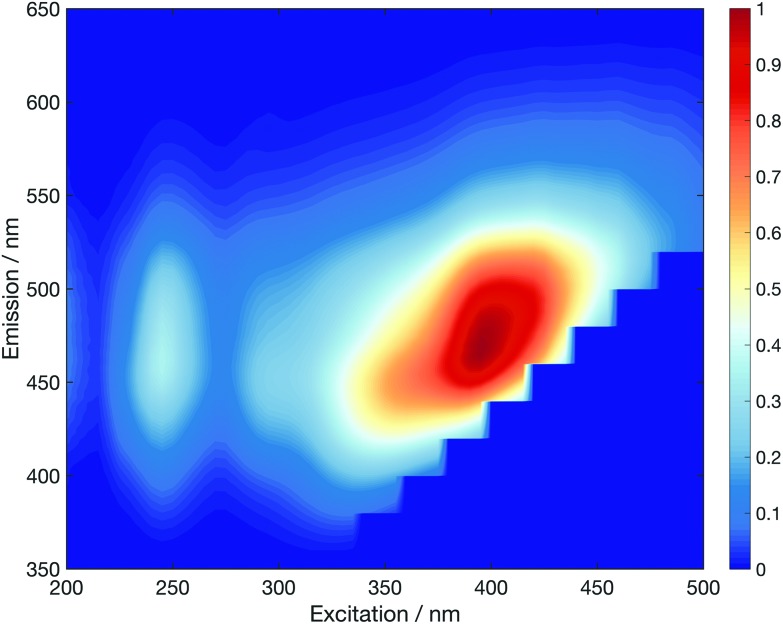
Normalised fluorescence excitation–emission spectrum for **5b** CDs.

Excitation between 340–365 nm, however, reveals a blue-shifted fluorescence maximum by up to ∼30 nm. This so-called “dual fluorescence” has recently been reported for similar CDs.^31^,^45^ Further, we note that the dual fluorescence is least pronounced for unfunctionalised CDs and obey Kasha's rule: the rate of internal conversion and vibrational/phonon energy relaxation is far greater than radiative rate which means fluorescence dominantly occurs from the lowest electronically excited energy level. This observation further bolsters our argument that glycan functionalisation significantly changes the electronic structure of the aromatic CD surface domains, which dominate the absorption spectra, at *λ* > 350 nm.

Ultrafast transient absorption spectroscopy (TA) was used to investigate the excited states lifetimes of the bare and carbohydrate functionalised CDs. Details of the experimental apparatus are given in the ESI.† Samples were photo-excited with 340 nm laser pulses and probed with a white light supercontinuum. TA spectra were recorded for many pump–probe time delays, *t*, between 0 and 1000 ps.

Fig. 3 displays TA spectra for the core CDs recorded at *t* = 1.5, 50 and 600 ps. The TA spectra are dominated by a positive excited state absorption (ESA) transient signal, which arises from electronic transitions between the initially photo-excited CD states and higher lying states. These TA spectra are reminiscent of those previously measured for other CDs synthesised in a microwave at ambient pressure.^31^ The small negative signal centred at ∼350 nm coincides with a peak in the linear absorption spectrum and corresponds to ground state bleaching (GSB) resulting from a reduced number of ground state CDs. From the linear absorption spectrum (Fig. S44†), we expect this to span down to 650 nm. The main spectral evolution observed in TA spectra consists of a blue-shift in the maxima signal intensity from ∼580 nm to ∼420 nm, due to phonon/vibrational relaxation, driven by interactions between the solvent and surface of the CDs. TA datasets for all CD species are shown in Fig. S47.†

**Fig. 3:**
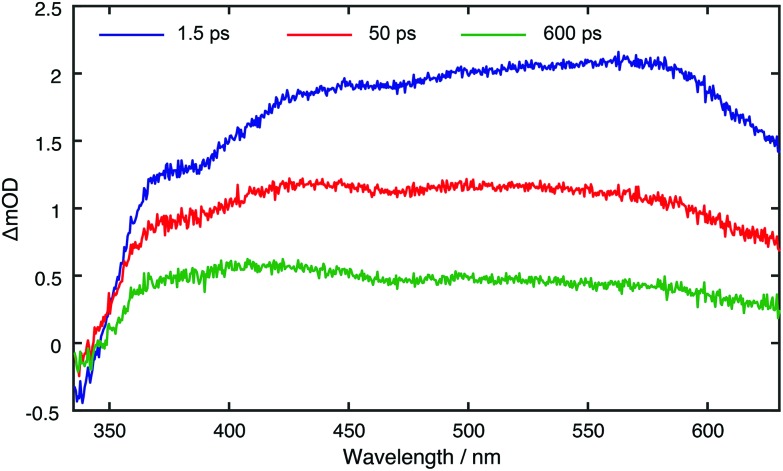
Core CD TA spectra for displayed pump–probe time delays.

Global analysis of the TA data was used to extract the spectral evolution of the overlapping transient features. The best fits to experimental data required a sequential kinetic scheme and three decay associated spectra (DAS). For full details see ESI.† The lifetimes returned from global analysis are summarised in Table 1. The third and longest lifetime component, *τ*_3_, is on the order of several nanoseconds and consistent with previously reported fluorescence lifetimes for a range of carbon dots synthesised *via* different methods.^7^,^31^–^33^,^45^,^46^

**Table 1:** Lifetimes for each DAS returned from global analysis of TA data, associated root mean square (RMS)

CD	*τ*_1_/ps	*τ*_2_/ps	*τ*_3_/ns	RMS/10^–5^
Core	5.4 ± 0.1	54.6 ± 0.4	1.22 ± 0.01	3.40
Glucose	11.0 ± 0.3	71.0 ± 2.0	1.5 ± 0.1	4.50
Mannose	4.2 ± 0.1	45.1 ± 0.3	1.12 ± 0.01	3.65
Galactose	13.6 ± 0.2	116.0 ± 5.0	1.5 ± 0.2	4.00
Maltose	15.0 ± 1.0	115.0 ± 4.0	10.0 ± 3.0	4.59
Lactose	4.5 ± 0.2	32.5 ± 0.6	1.27 ± 0.01	4.80

The shortest time constant, *τ*_1_, reported for unfunctionalised CDs is commensurate with the solvent re-organisation lifetime of methanol,^47^ which is required to mediate phonon/vibrational relaxation in CDs. There is, however, seemingly no obvious correlation between the chemical composition of the conjugated sugar, height of the surface functionalisation and the value of *τ*_1_. We recognise that our TA measurements are intrinsically an ensemble measurement unlike AFM measurements, and the variation in *τ*_1_ reflects the average solvent access to the CD aromatic layers which are screened by an inhomogeneous layer of conjugated glycans. We assume that the crystalline sp^3^ part of the core has no direct access to the solvent, and phonon relaxation must occur *via* coupling to the aromatic surface molecules and thence solvent and explains the positive correlation between the magnitude of the 100 s of picosecond (*τ*_2_) time constant with *τ*_1_. Conversely, *τ*_3_ is consistently around 1 ns, with the notable exception of maltose. Given that 340 nm light can excite both the sp^3^ crystalline and shell parts of the core (see ESI†), we assign the two picosecond time constants to multistep core relaxation, and the nanosecond component to surface domains where trapping may occur, and is likely responsible for the majority of the fluorescence.^32^,^33^

## Conclusions

In summary, we have demonstrated the successful conjugation of a range of carbohydrates to CDs, which have previously been shown to increase the biocompatibility and applicability of nanoparticles for the study of intracellular processes.^28^ We reveal that counter to prior reports, surface functionalisation of CDs does not necessarily result in a functionalised corona that is homogeneous, and the measured phonon/vibrational relaxation rates act as a probe of the ensemble average surface coverage. Our studies enable us to assign absorption bands associated with the crystalline core or aromatic shell states. Further, we demonstrate that the choice of carbohydrate functionalisation can dramatically alter the electronic structure of the surface states.

## Conflicts of interest

There are no conflicts to declare.

## Statement of contributions

T.A.S., M.D., R.L.H., S.A.H., D.B.-A., S.S., S.A.D and A.M.S. performed the experiments. T.A.S., M.D., R.L.H., D.B.-A., S.S, S.A.D, A.M.S., M.C.G. and T.A.A.O analyzed and interpreted the data. All authors contributed to the manuscript. M.C.G., H.M.W., and T.A.A.O. supervised the project.

## Supplementary Material

Supplementary information
