# Supplementary material for: Surface functionalisation significantly changes the physical and electronic properties of carbon nano-dots
Source: Nanoscale. 2018 Jul 10;10(29):13908–12. doi: 10.1039/c8nr03430c (PMC6067106; doi:10.1039/c8nr03430c)
Supplement: Supplementary file 1 [file NR-010-C8NR03430C-s001.pdf]

Electronic Supplementary Material (ESI) for Nanoscale.
This journal is © The Royal Society of Chemistry 2018

Supporting Information for:

Surface Functionalisation Significantly Changes the Physical and Electronic Properties of Carbon Nano-dots

Thomas A. Swift,[1,2,3] Marta Duchi,[1] Stephen A. Hill,[1] David Benito-Alifonso,[1] Robert L. Harniman,[1] Sadiyah Sheikh,[1,3] Sean A. Davis,[1,3] Annela M. Seddon,[3,4] Heather M. Whitney,[2,3] M. Carmen Galan[1,3*] and Thomas A.A. Oliver[1,3*]

[1] School of Chemistry, Cantock's Close, University of Bristol, BS8 1TS, UK

[2] School of Biological Sciences, Life Sciences Building, Tyndall Avenue, University of Bristol, BS8 1TQ, UK

[3] Bristol Centre for Functional Nanomaterials, HH Wills Physics Laboratory, Tyndall Avenue, University of Bristol, BS8 1TL, UK

[4] School of Physics, HH Wills Physics Laboratory, Tyndall Avenue, University of Bristol, BS8 1TL, UK

*Corresponding authors: tom.oliver@bristol.ac.uk, m.c.galan@bristol.ac.uk

GENERAL 3
Microwave assisted Kochetkov's amination *3*
Core synthesis *3*
Glycan functionalisation *3*
CHOICE OF CARBOHYDRATES 4
CD STABILITY 4
NMR SPECTROSCOPY 5
FOURIER TRANSFORMED INFRARED SPECTROSCOPY (FTIR) 16
GLYCAN-FUNCTIONALISATION QUANTIFICATION BY THE PHENOL-SULFURIC ACID ASSAY 19
TRANSMISSION ELECTRON MICROSCOPY (TEM) 20
SMALL ANGLE X-RAY SCATTERING (SAXS) 28
GUINIER ANALYSIS 28
ATOMIC FORCE MICROSCOPY (AFM) 31
ABSORPTION SPECTROSCOPY 38
FLUORESCENCE SPECTROSCOPY 39
TRANSIENT ABSORPTION DATA 42
GLOBAL ANALYSIS 43
ZETA POTENTIAL 44
REFERENCES 45

General

Chemicals were purchased and used without further purification. Core CD formation was conducted in a domestic microwave (Tesco Homebrand) 700W. Concentration centrifugation tubes were GE Healthcare Life Sciences VIVASPIN 20 with a 10 kDa molecular-weight cut off (MWCO) filter. Kochetkov's amination of carbohydrates was conducted using Biotage Initiator+ microwave reactor. 0.5-01 kDa MWCO Biotech Cellulose Ester membranes were used for dialysis. Extracts were concentrated under reduced pressure using both a Büchi rotary evaporator at a pressure of either 15 mmHg (diaphragm pump) at room temperature. ^{1}H and ^{13}C HSQC NMR were measured in D_2O at 500 MHz. All spectra were taken on a Bruker Advance III HD 500 Cryo. ^{1}H and ^{13}C NMR chemical shifts are quoted in parts per million (ppm) and referenced to the residual solvent peak (D_2O: 1-H = 4.70 ppm). Coupling constants (J) given in Hertz. Multiplicities are abbreviated as: s (singlet), d (doublet), t (triplet), q (quartet), p (pentet) and m (multiplet). Assignments were made with the aid of HSQC NMR experiments. FTIR was conducted on a Bruker ATR. Zeta analysis was carried out using Malvern Instruments Nano-Z ZEN 2600. Fluorescence measurements were taken on a Perkin-Elmer LS45 in quartz cuvettes purchased from ThorLabs. Absorbance measurements were conducted on Cary UV-Vis 50 spectrophotometer in quartz cuvettes. All measurements were made in HPLC-grade methanol. Before any of analysis the CDs were sonicated for a minimum of 30 minutes. A single batch of CD cores was used for all experiments. All data analysis was conducted using Matlab unless otherwise stated.

Microwave assisted Kochetkov's amination

Anhydrous DMSO (0.8 mL), ammonium carbonate (1g) and sugar (250 mg) were mixed together in a microwave vial and sealed. The vial was then heated to 60˚C for 90 minutes in a reaction microwave (10 W, 250 psi). The resulting solid was dissolved in H_2O and freeze dried.

Core synthesis

Glucosamine•HCl (1.00 g) and 4,7,10-trioxa-1,13-tridecanediamine (TTDDA) (1.35 ml) were mixed with 20 ml double-distilled water (ddH_2O). This mixture was then heated in a domestic microwave (3 mins, 700 W). The resulting brown oil was washed and sonicated with $CHCl_3$ several times, discarding the supernatant, until the supernatant is clear. The brown oil was then dissolved in 20 ml ddH_2O and centrifuged through GE Healthcare Life Sciences VIVASPIN 20 with a 10,000 Da MWCO filter (5000 rpm, 1 hour). The sample was then passed through a 200 nm syringe filter and dialysed overnight against ddH_2O at 0.5-01 kDa MWCO with Biotech Cellulose Ester membranes, replacing the ddH2O after the first couple of hours. The resulting solution was then freeze-dried to yield approximately ~1 g of core carbon dots.

Glycan functionalisation

The carbon dots were dissolved in methanol to a concentration of 10 $mg.mL^{-1}$ and sonicated. This solution was then passed through a 200 nm syringe filter and mixed with 0.5 equivalence by weight of succinic anhydride. The solution was subsequently stirred vigorously overnight. The resulting solution was then reduced and the brown oil washed and sonicated with tetrahydrofuran several times, discarding the supernatant, until the supernatant was clear. The resulting brown oil was then dissolved in methanol, reduced in *vacuo* and weighed. The brown oil was then dissolved in ddH_2O to a concentration of 10 $mgmL^{-1}$ and sonicated for 5 minutes. This solution was then passed through a 200 nm syringe filter and mixed with 2 equivalents by weight of

carbonyldiimidazole (CDI) and either 10 equivalents by weight of aminated monosaccharide or 20 equivalents by weight of aminated disaccharide to ensure homogeneous glycan functionalisation. This solution was then stirred vigorously overnight at room temperature. The solution was then freeze dried and the resulting brown oil was dissolved in ddH_2O and dialysed overnight against ddH_2O using Biotech Cellulose Ester membranes (at 0.5-01 kDa MWCO), replacing the ddH_2O after the first couple of hours. The resulting brown oil was then again freeze-dried. For storage the carbon dots were dissolved in either HPLC-grade methanol and kept at 4 ˚C to prevent aggregation.

The different glycan functionalisations were performed on the same batch of acid coated CDs, with all glycan conjugations performed with an excess of 1-aminoglycoside to ensure all the acid groups reacted. This ensured that any difference observed in the homogeneity of glycan corona after glycan conjugation is attributed to the nature of the different glycans.

Choice of Carbohydrates

Carbohydrates were chosen for their biological relevance and applications, as discussed in the main manuscript. A range of monosaccharides and disaccharides were used to explore the effect of different hydrophobic and hydrophilic regions formed by the different orientation of hydroxyl functional groups, although no trends emerged from our studies. Other carbohydrates such as trisaccharides (e.g. maltotriose) or polysaccharides (e.g. dextran) were not used to functionalised the CDs due to their aggregation in water and their low solubility in less polar solvents.

Carbohydrate-carbohydrate[1] and carbohydrate-protein[2] interactions have been suggested as mediators of important biological processes such as cell adhesion and aggregation, etc. These interactions are rather weak in a monovalent setting, but become significant when in a multivalent environment. Fundamental studies in the field to try to understand the nature of these key interactions have shown that carbohydrates can interact via hydrogen bonding through the OH groups and the apolar face in the sugar scaffold can also facilitate CH-π stacking interactions with aromatic motifs. [3,4] These interactions are dependant on the type of glycan and the glycan 3D surface presentation. It could be proposed that different glycans can thus interact with the surface of the CD they are conjugated on, which bears sp^2 motifs,[5] in a different manner depending on the nature of the sugar. Also, interactions between each class of glycan within each CD will be different depending on the sugar and therefore their 3D presentation would be affected.

CD stability

The core-CDs were also investigated by silica chromatography and size-exclusion chromatography (G10 Sephadex, Sigma). In both cases fluorescence and nanoscale structure could not be separated (observed by fluorescence spectroscopy and TEM). Previous work has also shown that the CDs are stable in a range of pH from 0-13 and when exposed UV light for a prelonged period.[5]

NMR spectroscopy

The ^{1}H and HSQC 500MHz NMR spectra of the CDs are shown below in figures S1-S6. All samples were dissolved at 5 mg/mL in 0.8 mL of D_2O using Norrell Select Series 7" NMR tubes (S-5-500-7). All spectra were taken on a Bruker Advance III HD 500 Cryo. All shifts are quoted in ppm. Residual internal D_2O is at δ= 4.70. Peaks were identified using both ^{1}H and HSQC NMR.

The ^{13}C and ^{1}H NMR spectra of the core CDs shown in Figure S1, and reproduce our previously reported data. [5] The peaks arising from the TTDDA linker are identified and assigned. Additional peaks to the TTDDA are also observed at the following ranges: hydrogen-1 δ= 7-9 and C-13 δ= 115-150. These peaks are produced by hydrogens on aromatics and phenols on the surface of the CDs. C=C bending and stretching, Aryl C-O stretching and a weak phenol O-H bending signal can also be observed in the FTIR to support this conclusion.

The NMR spectra for the glycan-functionalised CDs are shown in figures S2-S6. For each of these spectra the TTDDA linker is identified, although often not completely assigned due to overlapping peaks from the functionalised carbohydrates (hydrogens attached to carbons 2-6). These peaks are themselves also difficult to assign individually due to their overlap, however appear in the following characteristic ranges: ^{1}H δ= 3.0-3.8 and C-13 δ= 55-80. Due to the apparent congestion in the NMR spectra, the successful carbohydrate functionalisation was instead determined by the identification of the characteristic doublet peak associated with the hydrogen on the anomeric carbon of intact carbohydrates and which is shifted when compared to the unconjugated glycoside. This doublet is found in the following ranges: ^{1}H δ= 4.2-5.5 and ^{13}C δ= 90-105. Often more than one doublet is observed, which arises from reduced conformational freedom that the carbohydrate experiences due to being tethered to the nanoparticle surface. This was observed for all of the glycan-functionalised CDs, and agrees with our previous NMR studies of lactose-CDs and work on the glycan-functionalisation of CdSe quantum dots. [5,6] For each of the CD species, peak assignments are given in the figure caption and labelled with reference to the respective skeletal CD-glycan structures.

To further confirm successful glycan functionalisation of the nanoparticles, Diffusion-Ordered NMR Spectroscopy (DOSY), which probes the diffusion coefficient for each of the components of the ^{1}H NMR spectrum, was acquired. The anomeric peaks of each of the sugars were observed to have similar diffusion coefficients to the chemical shifts associated with the CD core, confirming that the glycosides are attached to the CDs. The DOSY data with their accompanying ^{1}H NMR spectra are shown in figures S7-S12. The DOSY data was analysed using DOSY Toolbox produced by Nilsson *et al.*. [7] The effective hydrodynamic radius of the CDs is also calculated from the diffusion coefficient using the Stokes-Einstein equation:

$$D = \frac{k_B T}{6\pi\eta r} \tag{1}$$

Where D is the diffusion coefficient, k_B is the Boltzmann constant, T is the temperature, η is the dynamic viscosity and r is the effective hydrodynamic radius of the particle. This should not be considered as an accurate measure of particle diameter as the particle is assumed to be spherical.

All of the DOSY peaks corresponding to the glycan functionalisation on the CDs were observed to have smaller diffusion coefficients than free sugars in solution therefore are attached to a particle of larger volume. A table of literature values of the diffusion coefficients for carbohydrates as well as the values for the DOSY peaks associated with the glycan-CDs are given in Table S1 for comparison.

Table S1. *The obtained glycan-CD diffusion coefficients are highlighted in red, these are taken from the average value for the hydrogen of the anomeric carbon of the glycan functionalisation of the CDs. If temperature of measurement is given for literature values the values quoted were measured at 298K.*

**Carbohydrate or Glycan-CD**	**Diffusion Coefficient ($\times 10^{-10}$ m^2 s^{-1})**	**Reference or Figure Number**
D-Glucose	6.3	Ref. [8]
α-D-glucose	7.6	Ref. [9]
β-D-glucose	5.8	Ref. [9]
Glucose-CD	4.6	Fig. S8
D-Mannose	7.0	Ref. [10]
α-D-Mannose	7.4	Ref. [9]
β-D-Mannose	6.85	Ref. [9]
Mannose-CD	4.2	Fig. S9
D-galactose	8.7	Ref. [8]
α-D-galactose	10.9	Ref. [9]
β-D-galactose	5.8	Ref. [9]
Galactose-CD	4.1	Fig. S10
Maltose	5.2	Ref. [11]
Maltose-CD	3.4	Fig. S11
Lactose	5.6	Ref. [12]
Lactose-CD	4.1	Fig. S12

The ratios of the components of the glycan-CDs was also investigated by NMR, this is shown in Figures S13-S14 and Table S2.

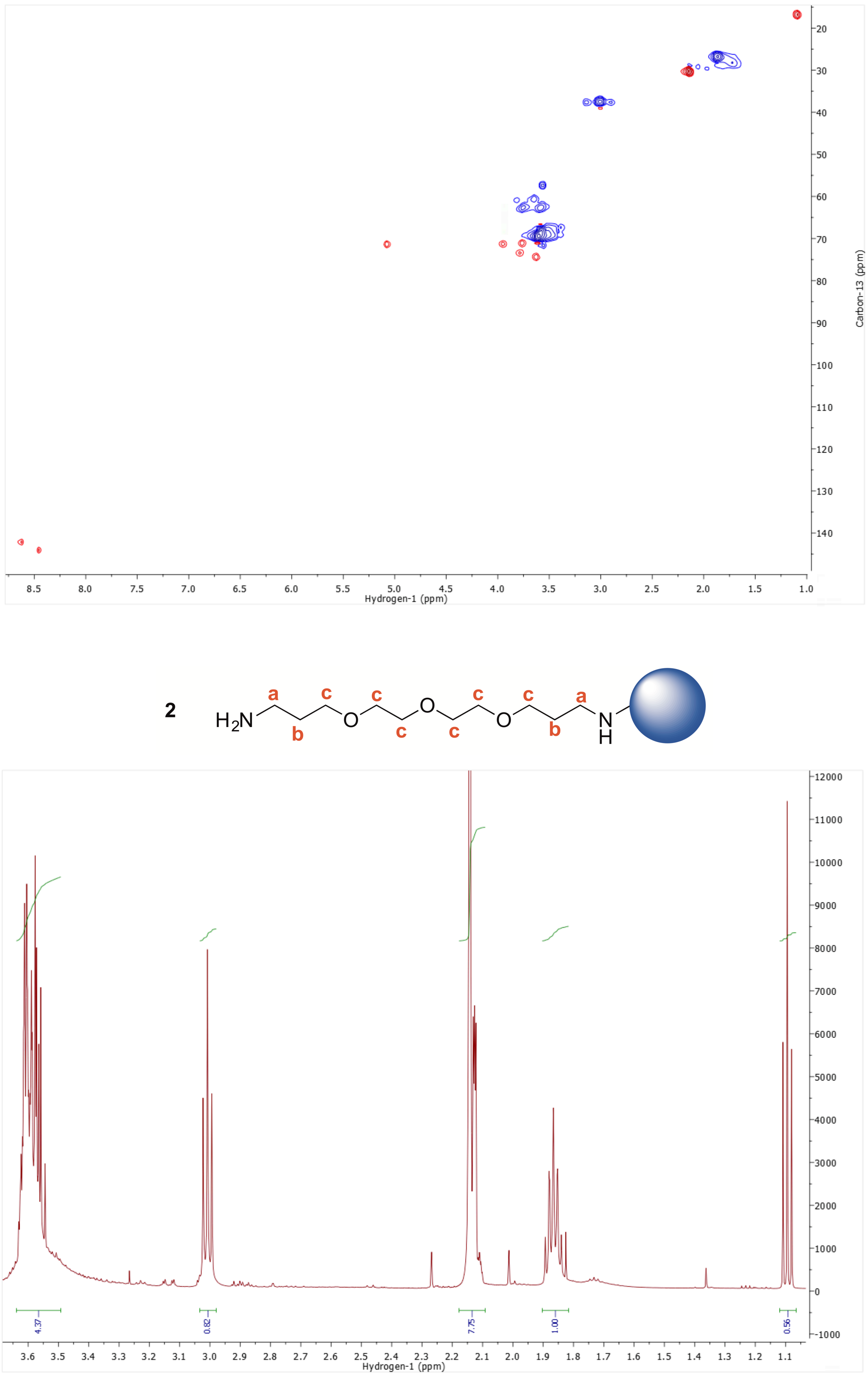

Figure S1: *The* ^{1}*H (bottom) and HSQC (top) NMR spectra of the core-CDs. δ: 3.68 – 3.49 (m, 16H, H-c), 3.01 (t, J = 7.2 Hz, 4H, H-a), 2.14 (d, J = 0.7 Hz), 1.93 – 1.81 (q, 4H, H-b), 1.09 (t, J = 7.1 Hz).*

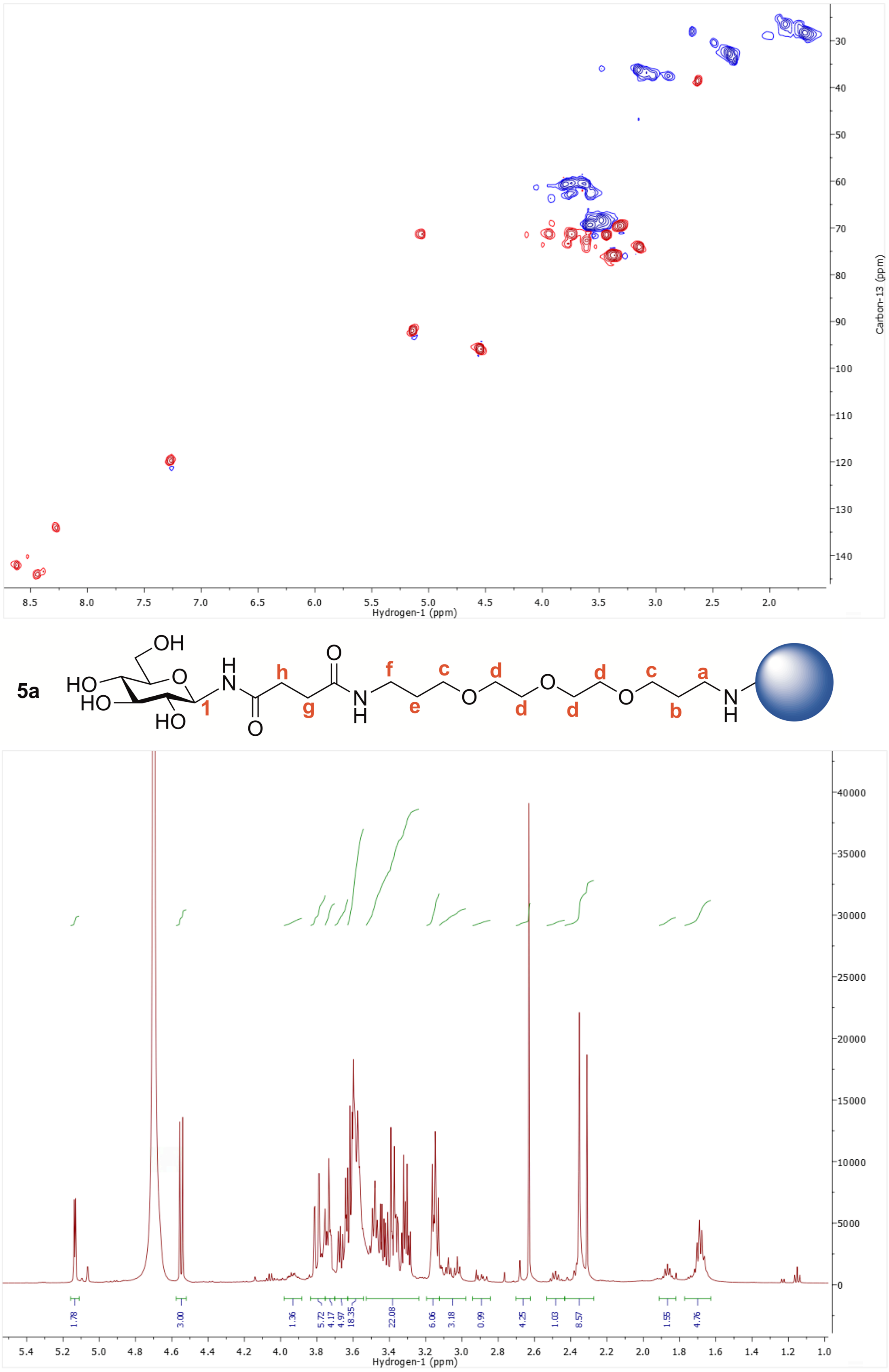

Figure S2: *The ^{1}H (bottom) and HSQC (top) NMR spectra of the glucose-CDs. δ: 5.14 (d, J = 3.8 Hz, 1H, H-1), 4.55 (d, J = 7.9 Hz, 1H, H-1), 3.58 – 3.25 (t, 12H, H-c, and H-d), 3.20 – 3.11 (t, 2H, H-f), 3.14 – 2.97 (t, 2H, H-a), 2.33 (t, J = 20.9 Hz), 1.91 – 1.79 (t, 2H, H-b), 1.69 (p, J = 6.6 Hz, 2H, H-e).*

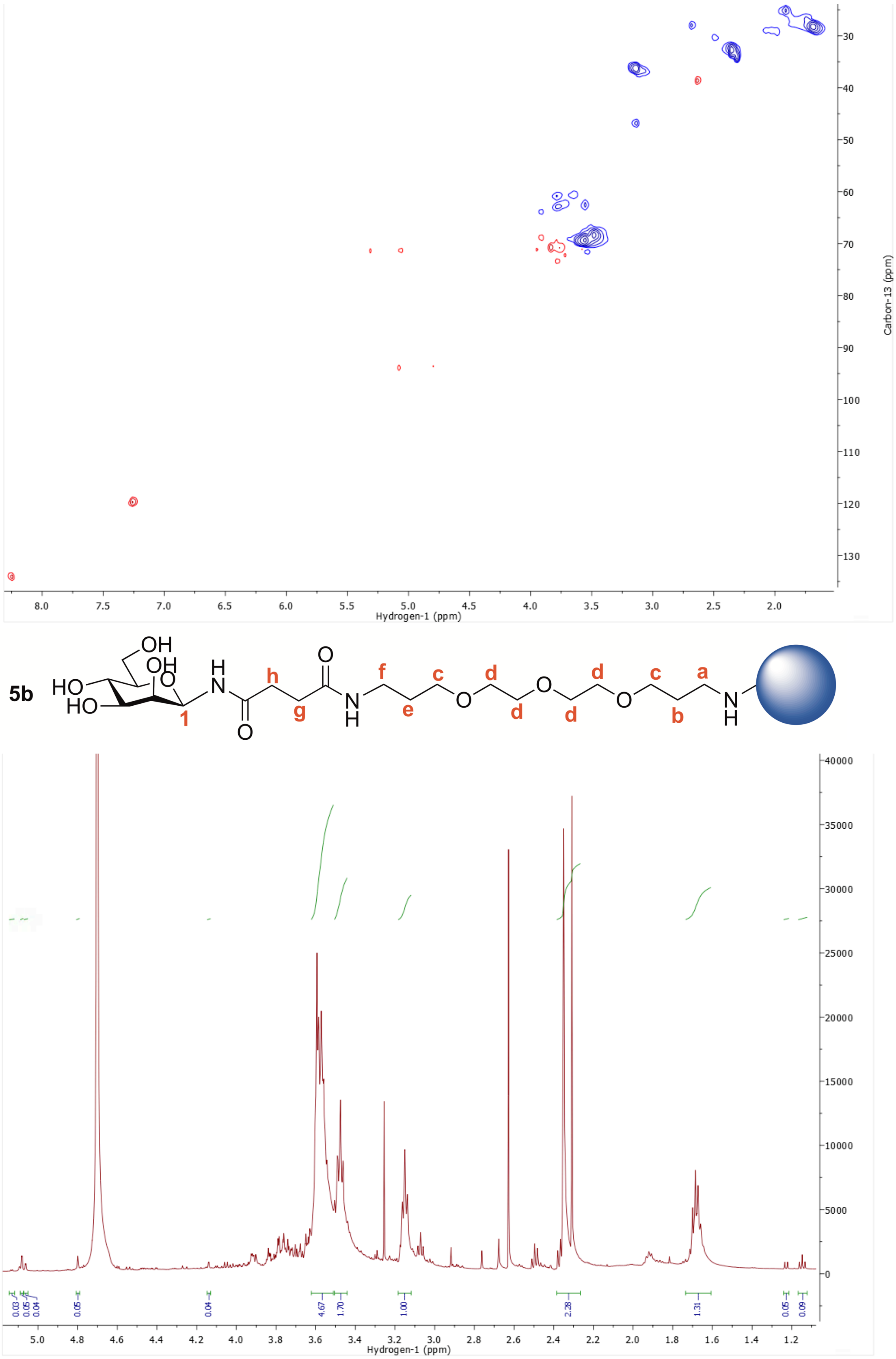

Figure S3: *The* ^{1}*H (bottom) and HSQC (top) NMR spectra of the mannose-CDs. δ: 5.13 (d, J = 3.8 Hz, 1H, H-1), 5.09 (d, J = 2.0 Hz, 1H, H-1), 5.08 (d, J = 1.7 Hz, 1H, H-1), 5.06 (d, J = 2.2 Hz, 1H, H-1), 4.80 (d, J = 1.1 Hz, 1H, H-1), 3.62 – 3.53 (m, 8H, H-d), 3.50 – 3.45 (t, 4H, H-c), 3.15 (t, J = 6.5 Hz, 2H, H-f), 3.07 (t, J = 6.7 Hz, H-a), 2.50 (t, J = 7.7Hz, 2H, H-h), 1.69 (p, 2H, H-e).*

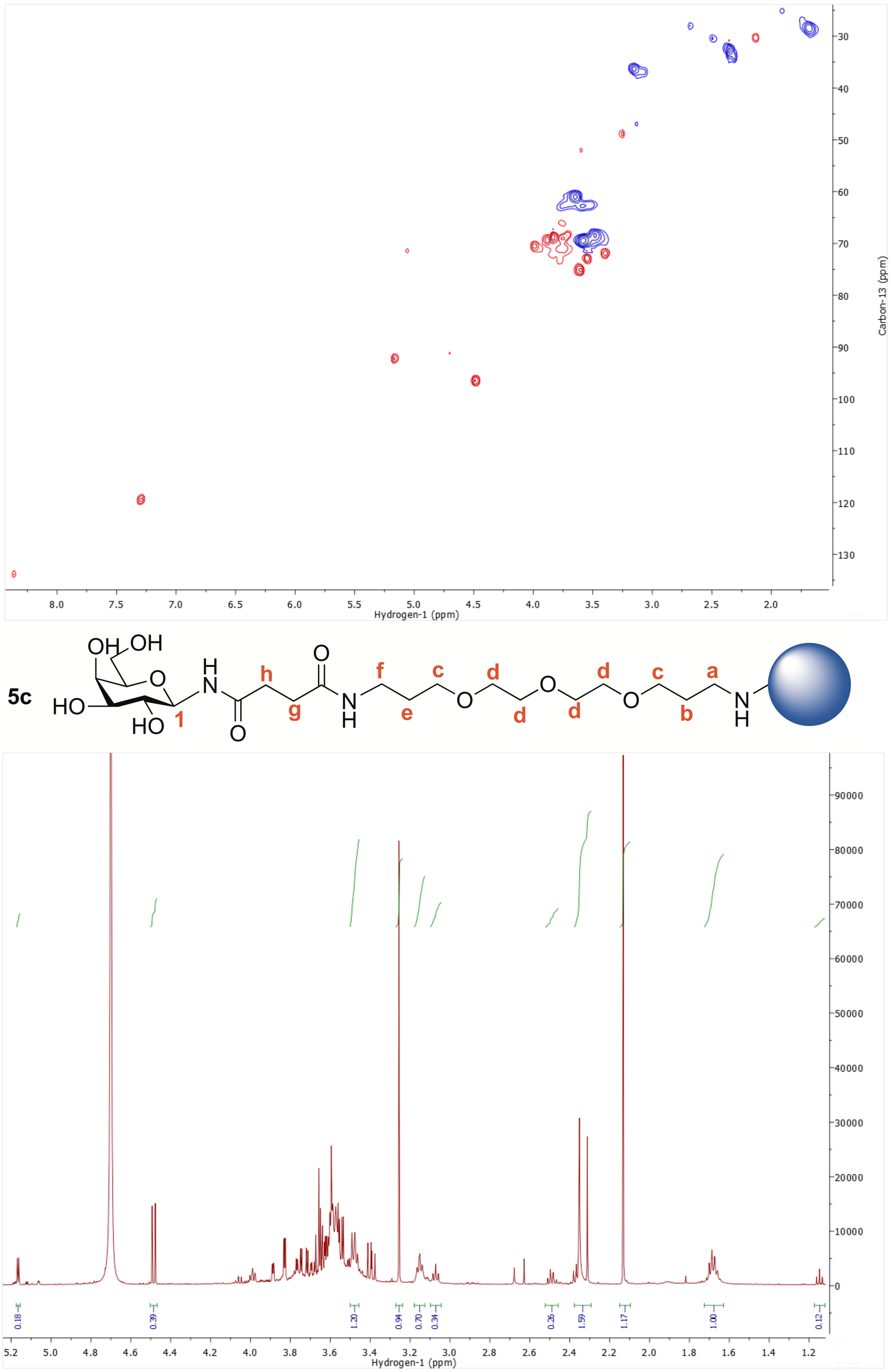

Figure S4: *The ^{1}H (bottom) and HSQC (top) NMR spectra of the galactose-CDs. δ: 5.17 (d, J = 3.7 Hz, 1H, H-1), 4.49 (d, J = 7.9 Hz, 1H, H-1), 3.71 – 3.45 (m, 8H, H-d), 3.47 – 3.31 (t, 4H, H-c), 3.20 – 3.03 (t, 2H, H-f), 2.53 – 2.41 (t, 2H, H-h), 2.42 – 2.31 (t, 2H, H-g), 1.77 – 1.62 (p, 2H, H-e).*

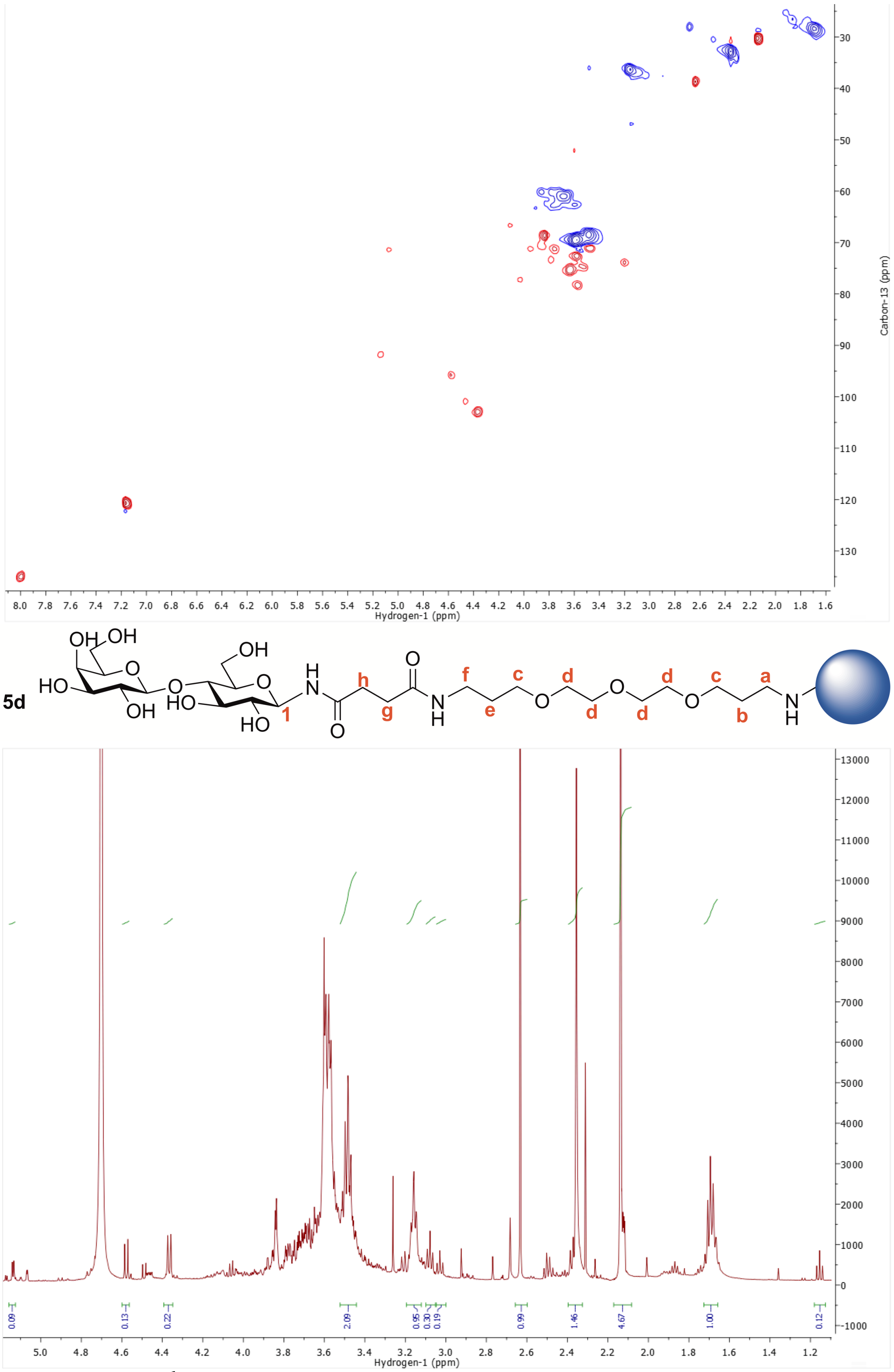

Figure S5: *The ^{1}H (bottom) and HSQC (top) NMR spectra of the lactose-CDs. δ: 5.32 (d, J = 3.9 Hz, 2H, H-1), 5.14 (d, J = 3.9 Hz, 2H, H-1), 4.58 (d, J = 8.0 Hz, 2H, H-1), 4.37 (d, J = 7.9 Hz, 2H, H-1), 3.72 – 3.49 (m, 8H, H-d), 3.48 (t, J = 6.6Hz, 4H, H-c), 3.08 (t, J = 6.8 Hz, 2H, H-f), 3.03 (t, J = 7.1 Hz, 2H, H-a), 2.52 – 2.48 (t, 2H, H-h), 1.89 – 1.85 (p, 2H, H-b), 1.71 – 1.65 (p, 2H, H-e)*

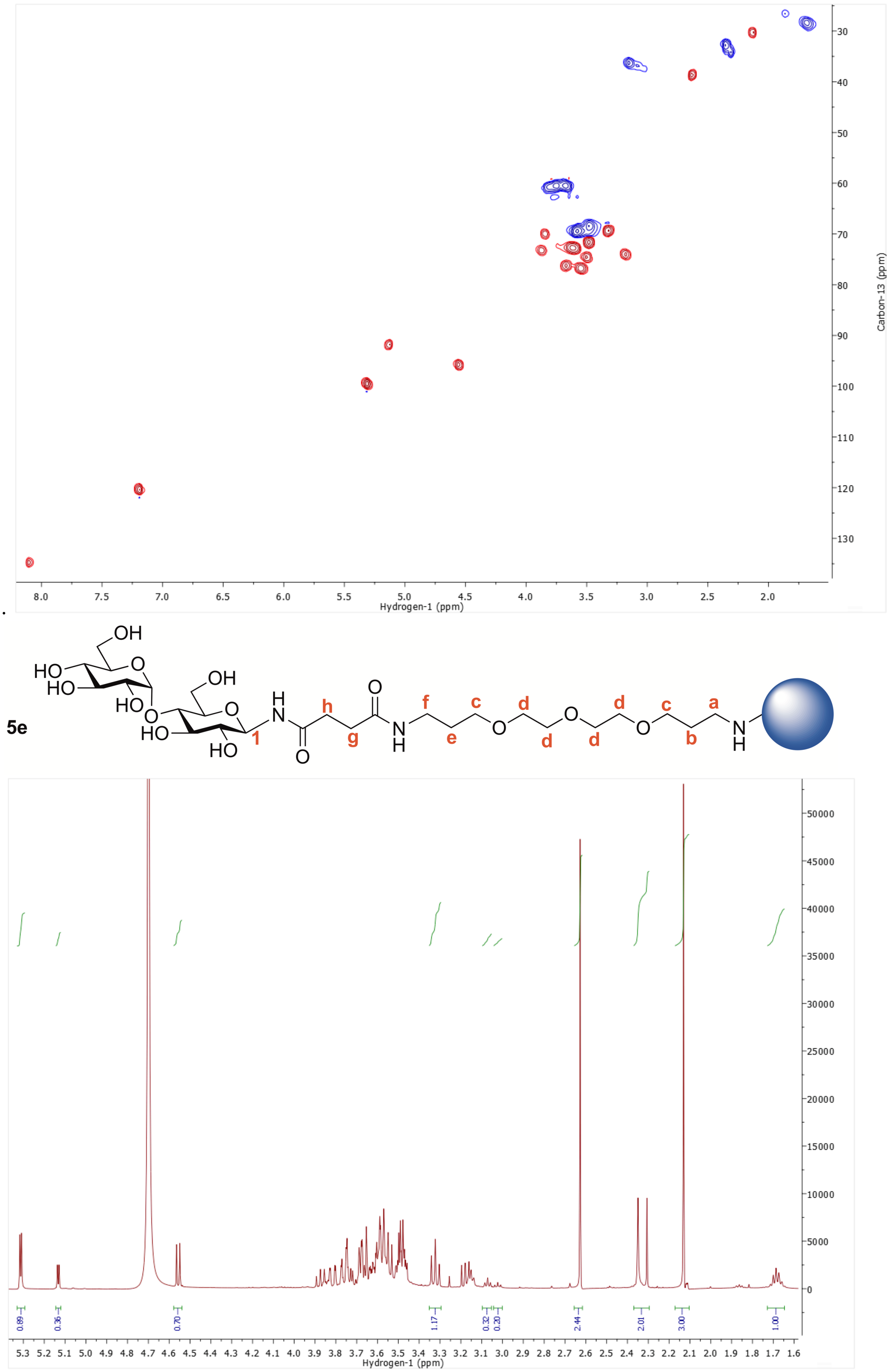

Figure S6: *The ^{1}H (bottom) and HSQC (top) NMR spectra of the maltose-CDs. δ: 5.31 (d, J = 3.9 Hz, 2H, H-1), 5.13 (d, J = 3.7 Hz, 2H, H-1), 4.56 (d, J = 7.9 Hz, 2H, H-1), 3.60 – 3.40 (m, 8H, H-d), 3.32 (t, J = 9.5 Hz, 4H, H-c), 3.22 – 3.12 (m, 2H, H-f), 2.33 (d, J = 21.5 Hz, 2H), 1.73 – 1.62 (p, 1H, H-b and H-e).*

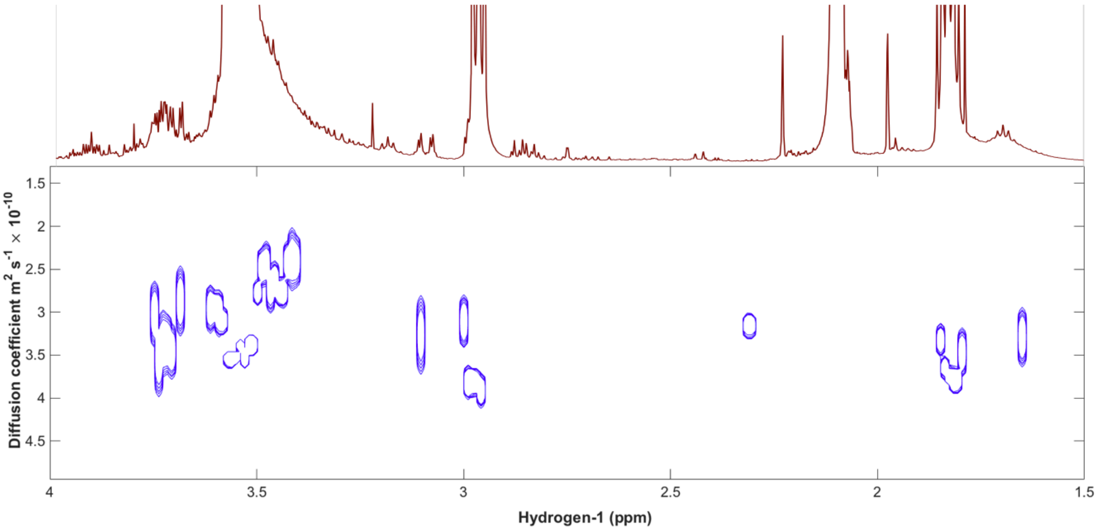

Figure S7: *The DOSY and ^{1}H NMR spectra of the core-CDs. The following ^{1}H shifts correspond to the core CD δ: 3.68 – 3.49 (m, 16H, H-c), 3.01 (t, J = 7.2 Hz, 4H, H-a), 2.14 (d, J = 0.7 Hz), 1.93 – 1.81 (q, 4H, H-b). All the peaks in the ^{1}H NMR spectra that correspond to the CDs have diffusion coefficients in the range (1.8 – 4.5) ×10^{-10} m^2.s^{-1} which corresponds to a particle radius of (0.55 – 1.36) nm.*

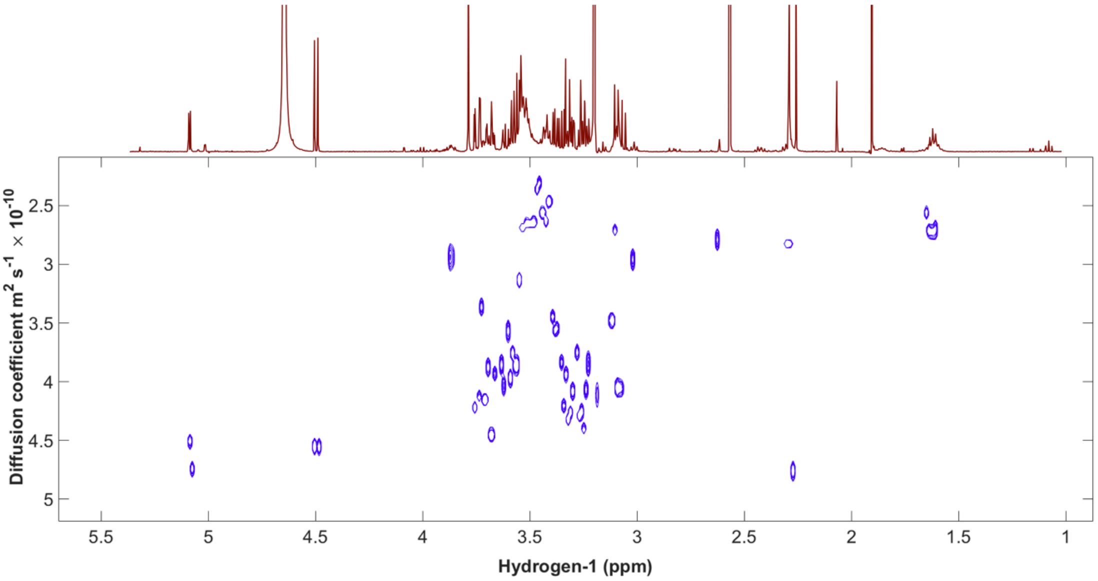

Figure S8: *The DOSY and ^{1}H NMR spectra of the glucose-CDs. The following ^{1}H shifts correspond to the core CD δ: 3.58 – 3.25 (t, 12H, H-c, and H-d), 3.20 – 3.11 (t, 2H, H-f), 3.14 – 2.97 (t, 2H, H-a), 2.33 (t, J = 20.9 Hz), 1.91 – 1.79 (t, 2H, H-b), 1.69 (p, J = 6.6 Hz, 2H, H-e), the following correspond to the glucose δ: 5.14 (d, J = 3.8 Hz, 1H, H-1), 4.55 (d, J = 7.9 Hz, 1H, H-1). All the peaks in the ^{1}H NMR spectra that correspond to the glucose-CDs have diffusion coefficients in the range (2.2 – 4.8)×10^{-10} m^2.s^{-1} which corresponds to a particle radius of (0.51 – 1.12) nm.*

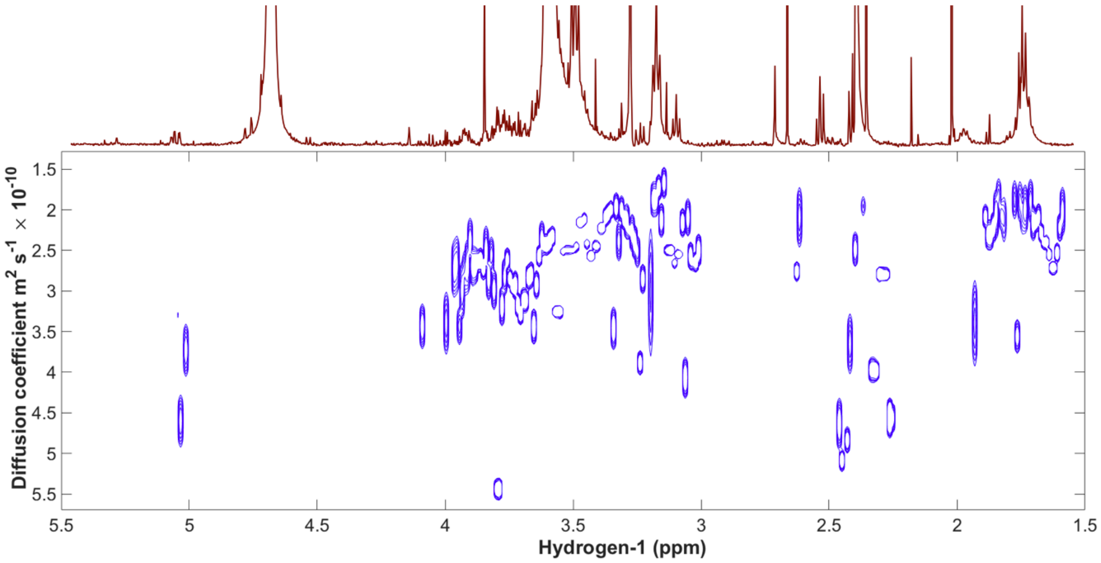

Figure S9: *The DOSY and ^{1}H NMR spectra of the mannose-CDs. The following ^{1}H shifts correspond to the core CD δ: 3.62 – 3.53 (m, 8H, H-d), 3.50 – 3.45 (t, 4H, H-c), 3.15 (t, J = 6.5 Hz, 2H, H-f), 3.07 (t, J = 6.7 Hz, H-a), 2.50 (t, J = 7.7Hz, 2H, H-h), 1.69 (p, 2H, H-e), the following correspond to the mannose δ: 5.13 (d, J = 3.8 Hz, 1H, H-1), 5.09 (d, J = 2.0 Hz, 1H, H-1). All the peaks in the ^{1}H NMR spectra that correspond to the mannose-CDs have diffusion coefficients in the range (1.5 – 5.2)×10^{-10} m^2.s^{-1} which corresponds to a particle radius of (0.48 – 1.64) nm.*

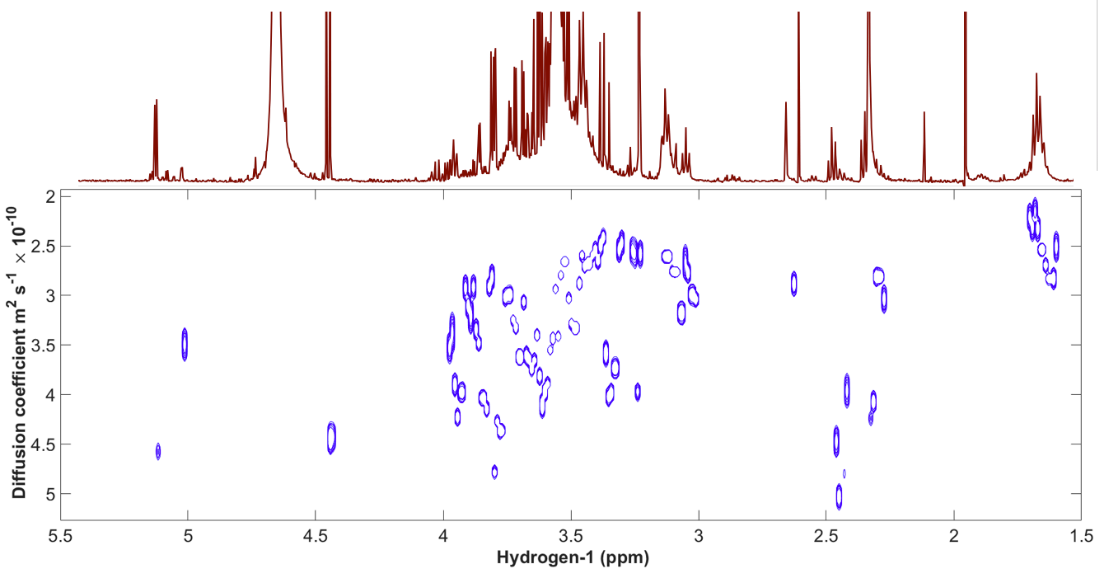

Figure S10: *The DOSY and ^{1}H NMR spectra of the galactose-CDs. The following ^{1}H shifts correspond to the core CD δ: 3.71 – 3.45 (m, 8H, H-d), 3.47 – 3.31 (t, 4H, H-c), 3.20 – 3.03 (t, 2H, H-f), 2.53 – 2.41 (t, 2H, H-h), 2.42 – 2.31 (t, 2H, H-g), 1.77 – 1.62 (p, 2H, H-e), the following correspond to the galactose δ: 5.17 (d, J = 3.7 Hz, 1H, H-1), 4.49 (d, J = 7.9 Hz, 1H, H-1). All the peaks in the ^{1}H NMR spectra that correspond to the galactose-CDs have diffusion coefficients in the range (1.5 – 5.2)×10^{-10} m^2.s^{-1} which corresponds to a particle radius of (0.48 – 1.64) nm.*

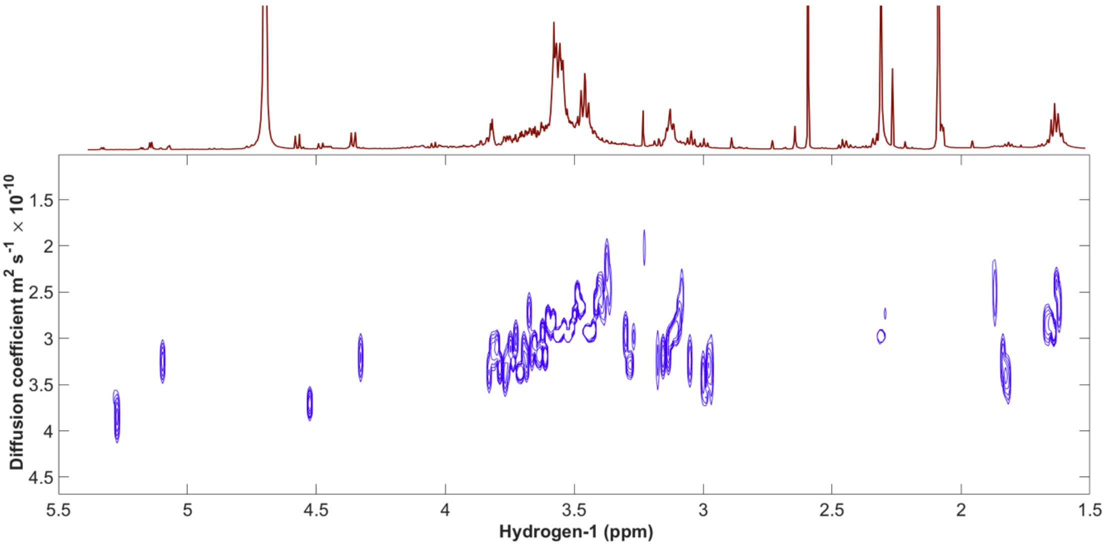

Figure S11: *The DOSY and ^{1}H NMR spectra of the lactose-CDs. The following ^{1}H shifts correspond to the core CD δ: 3.72 – 3.49 (m, 8H, H-d), 3.48 (t, J = 6.6Hz, 4H, H-c), 3.08 (t, J = 6.8 Hz, 2H, H-f), 3.03 (t, J = 7.1 Hz, 2H, H-a), 2.52 – 2.48 (t, 2H, H-h), 1.89 – 1.85 (p, 2H, H-b), 1.71 – 1.65 (p, 2H, H-e), the following correspond to the lactose δ: 5.32 (d, J = 3.9 Hz, 2H, H-1), 5.14 (d, J = 3.9 Hz, 2H, H-1), 4.58 (d, J = 8.0 Hz, 2H, H-1), 4.37 (d, J = 7.9 Hz, 2H, H-1). All the peaks in the ^{1}H NMR spectra that correspond to the lactose-CDs have diffusion coefficients in the range (1.8 – 4.2)$\times 10^{-10}$ $m^{2}.s^{-1}$ which corresponds to a particle radius of (0.58 – 1.36) nm.*

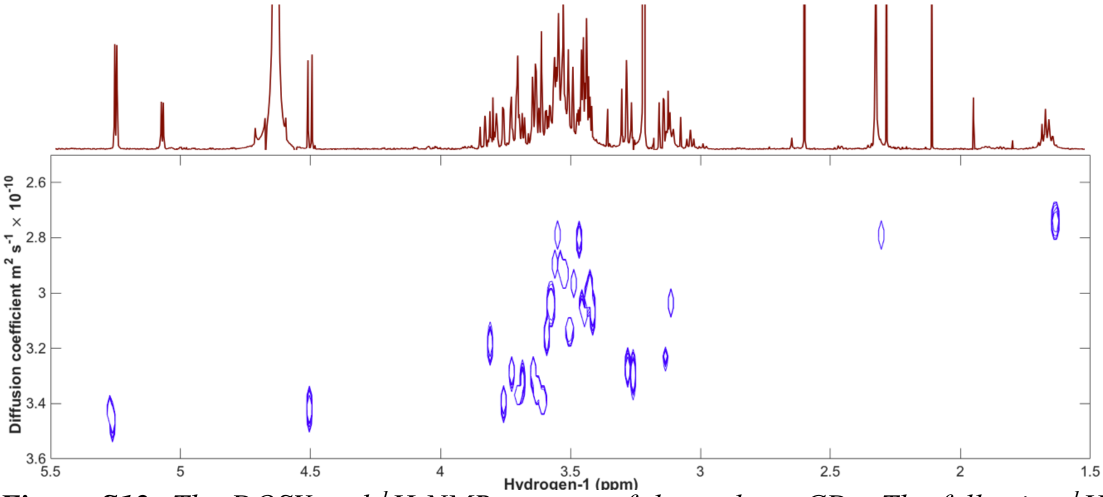

Figure S12: *The DOSY and ^{1}H NMR spectra of the maltose-CDs. The following ^{1}H shifts correspond to the core CD δ: 3.60 – 3.40 (m, 8H, H-d), 3.32 (t, J = 9.5 Hz, 4H, H-c), 3.22 – 3.12 (m, 2H, H-f), 2.33 (d, J = 21.5 Hz, 2H), 1.73 – 1.62 (p, 1H, H-b and H-e), the following correspond to the maltose δ: 5.31 (d, J = 3.9 Hz, 2H, H-1), 4.56 (d, J = 7.9 Hz, 2H, H-1). All the peaks in the ^{1}H NMR spectra that correspond to the maltose-CDs have diffusion coefficients in the range (2.6 – 3.6)$\times 10^{-10}$ $m^{2}.s^{-1}$ which corresponds to a particle radius of (0.68 – 0.94) nm.*

Fourier transformed infrared spectroscopy (FTIR)

The CDs were investigated using FTIR. The spectra support the earlier findings of NMR spectroscopy and shown in Figures S13-S18.

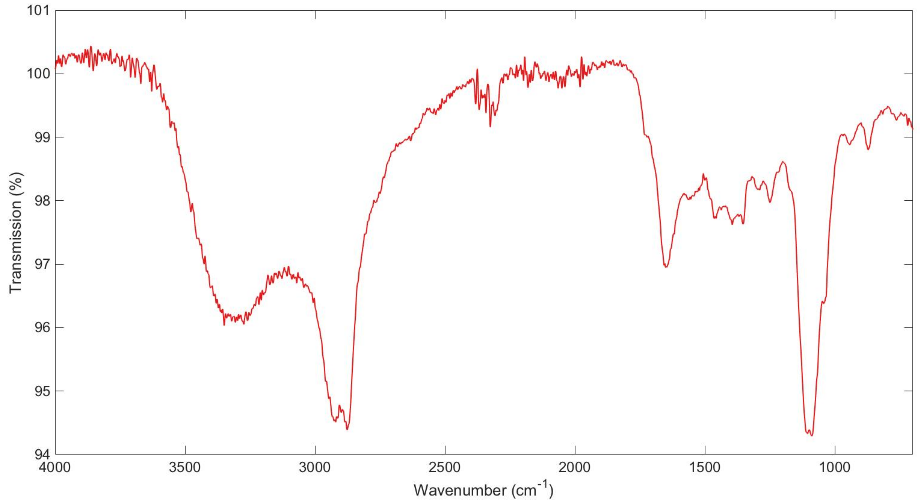

Figure S13: *Core-CDs, peaks identified as: 870cm^{-1} and 940cm^{-1} (C=C bending); 1045cm^{-1} (ether C-O stretching); 1090cm^{-1}and 1105cm^{-1} (primary and secondary alcohol C-O stretching); 1355cm^{-1}, 1395cm^{-1} and 1460cm^{-1} (alcohol O-H bending); 1555cm^{-1} (C=C stretching); 1650cm^{-1} (amide C=O stretching); 2875cm^{-1} and 2925cm^{-1} (sp^3 C-H stretching); 3300cm^{-1} (alcohol O-H or amine N-H stretching).*

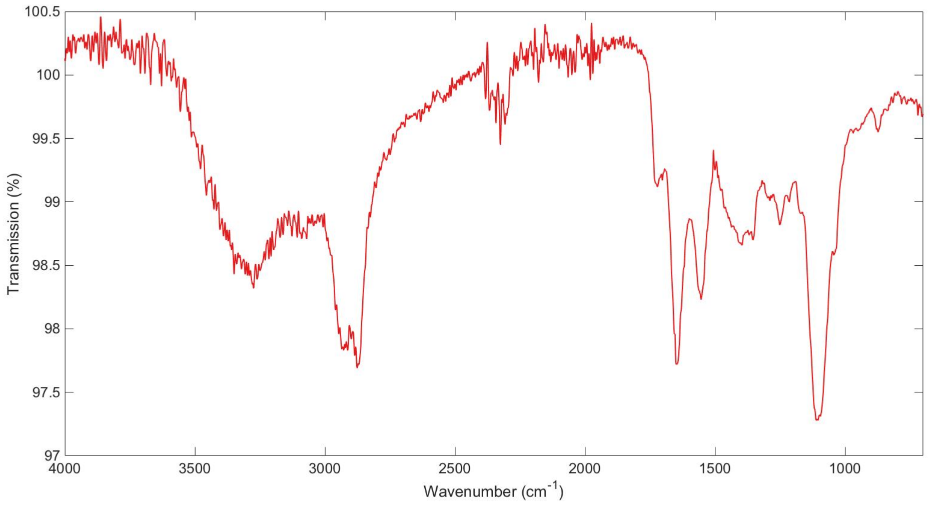

Figure S14: *Glucose-CDs, peaks identified as: 870cm^{-1} and 950cm^{-1} (C=C bending); 1040cm^{-1} (ether C-O stretching); 1095cm^{-1}and 1105cm^{-1} (primary and secondary alcohol C-O stretching); 1355cm^{-1} and 1400cm^{-1} (alcohol O-H bending); 1555cm^{-1} (C=C stretching); 1645cm^{-1} (amide C=O stretching); 1725cm^{-1} (carboxylic acid C=O stretching); 2875cm^{-1} and 2925cm^{-1} (sp^3 C-H stretching); 3300cm^{-1} (alcohol O-H or amine N-H stretching).*

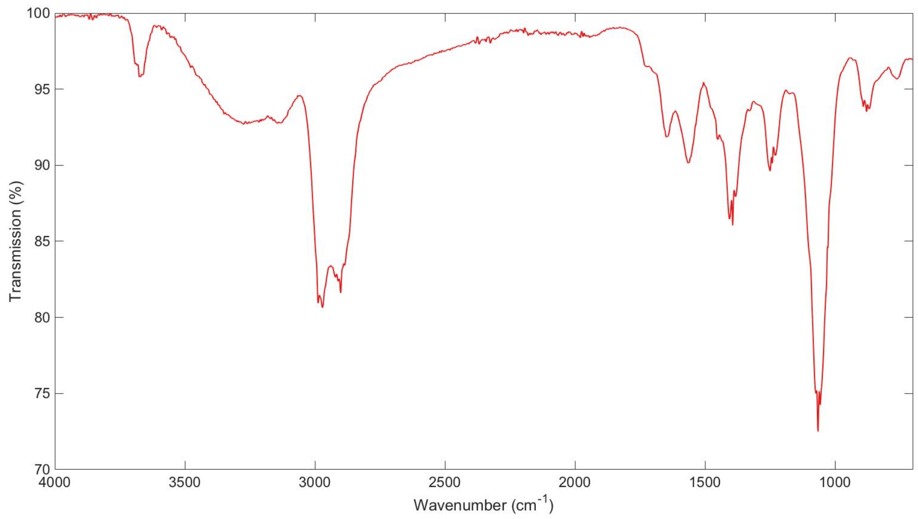

Figure S15: *Mannose-CDs, peaks identified as: 870cm^{-1} 880cm^{-1} and 890cm^{-1} (C=C bending); 1065cm^{-1} (primary and secondary alcohol C-O stretching); 1330cm^{-1} (possibly phenol O-H bending); 1385cm^{-1}, 1395cm^{-1} and 1410 cm^{-1} (alcohol O-H bending); 1450cm^{-1} (alkane C-H bending); 1565cm^{-1} (C=C stretching); 1645cm^{-1} (amide C=O stretching); 1730cm^{-1} (carboxylic acid C=O stretching); 2885cm^{-1}, 2900cm^{-1}, 2910cm^{-1}, 2920cm^{-1}, 2970cm^{-1} and 2990cm^{-1} (sp^3 C-H stretching); 3300cm^{-1} (alcohol O-H or amine N-H stretching); 3675cm^{-1} (alcohol O-H stretching).*

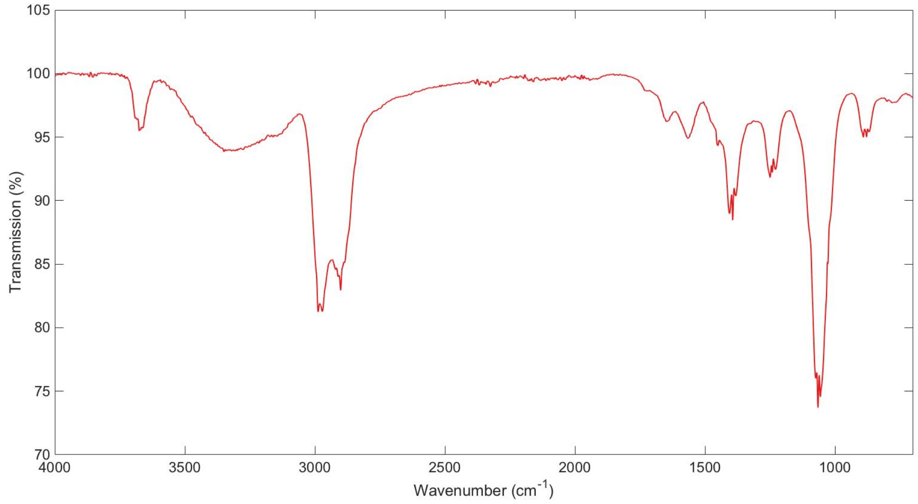

Figure S16: *Galactose-CDs, peaks identified as: 870cm^{-1} 880cm^{-1} and 890cm^{-1} (C=C bending); 1065cm^{-1} (primary and secondary alcohol C-O stretching); 1330cm^{-1} (possibly phenol O-H bending); 1385cm^{-1}, 1395cm^{-1} and 1405 cm^{-1} (alcohol O-H bending); 1450cm^{-1} (alkane C-H bending); 1565cm^{-1} (C=C stretching); 1645cm^{-1} (amide C=O stretching); 1730cm^{-1} (carboxylic acid C=O stretching); 2885cm^{-1}, 2900cm^{-1}, 2910cm^{-1}, 2920cm^{-1}, 2970cm^{-1} and 2990cm^{-1} (sp^3 C-H stretching); 3300cm^{-1} (alcohol O-H or amine N-H stretching); 3675cm^{-1} (alcohol O-H stretching).*

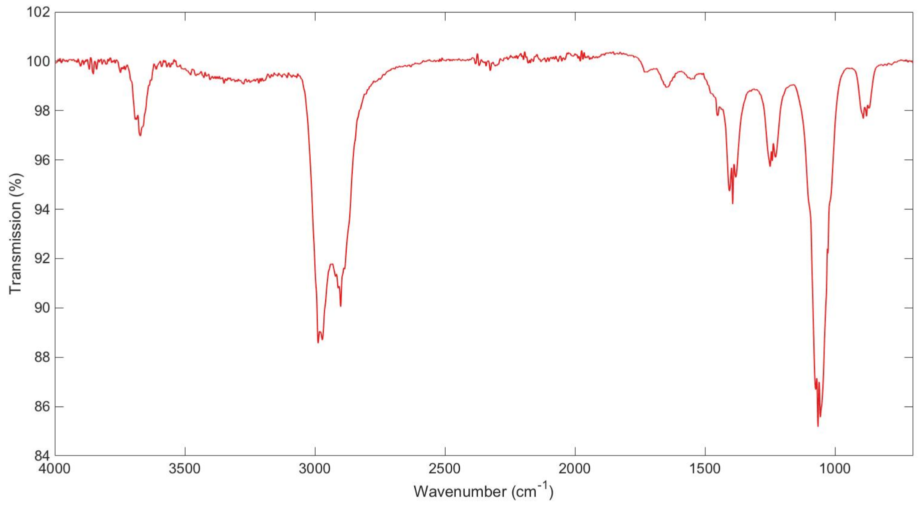

Figure S17: *Maltose-CDs, peaks identified as: 870cm^{-1} 880cm^{-1} and 890cm^{-1} (C=C bending); 1065cm^{-1} (primary and secondary alcohol C-O stretching); 1330cm^{-1} (possibly phenol O-H bending); 1385cm^{-1}, 1395cm^{-1} and 1410 cm^{-1} (alcohol O-H bending); 1450cm^{-1} (alkane C-H bending); 1560cm^{-1} (C=C stretching); 1650cm^{-1} (amide C=O stretching); 1725cm^{-1} (carboxylic acid C=O stretching); 2885cm^{-1}, 2900cm^{-1}, 2910cm^{-1}, 2920cm^{-1}, 2970cm^{-1} and 2990cm^{-1} (sp^3 C-H stretching); 3300cm^{-1} (alcohol O-H or amine N-H stretching); 3670cm^{-1}and 3690cm^{-1} (alcohol O-H stretching).*

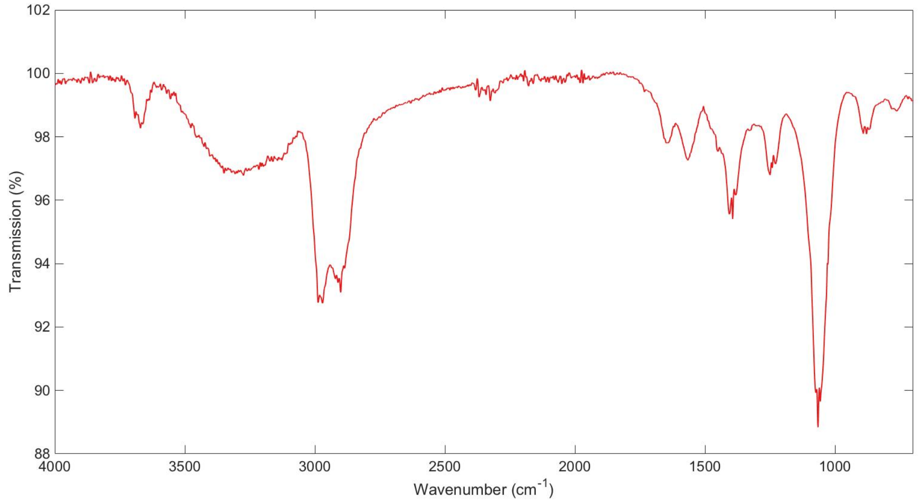

Figure S18: *Lactose-CDs, peaks identified as: 870cm^{-1} 880cm^{-1} and 890cm^{-1} (C=C bending); 1060cm^{-1}, 1065cm^{-1} and 1070cm^{-1} (primary and secondary alcohol C-O stretching); 1330cm^{-1} (possibly phenol O-H bending); 1385cm^{-1}, 1395cm^{-1} and 1410 cm^{-1} (alcohol O-H bending); 1450cm^{-1} (alkane C-H bending); 1565cm^{-1} (C=C stretching); 1650cm^{-1} (amide C=O stretching); 1725cm^{-1} (carboxylic acid C=O stretching); 2885cm^{-1}, 2900cm^{-1}, 2910cm^{-1}, 2920cm^{-1}, 2970cm^{-1} and 2990cm^{-1} (sp^3 C-H stretching); 3300cm^{-1} (alcohol O-H or amine N-H stretching); 3670cm^{-1}and 3690cm^{-1} (alcohol O-H stretching).*

Glycan-functionalisation quantification by the phenol-sulfuric acid assay

The glycan functionalisation was quantified using the phenol-sulphuric acid assay previously developed by others.[13] The calibration curve for the different carbohydrates is shown in Figure S19.

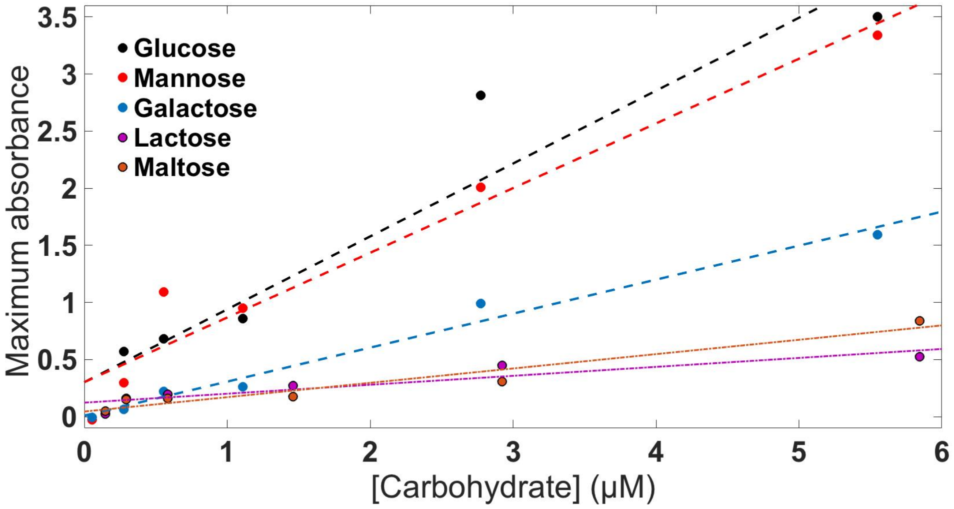

Figure S19: *The absorbance calibration plots for the assay. The concentration of carbohydrate refers to the concentration at which it was added to the mix. The peak absorbance was identified at 490nm for all the carbohydrates.*

The concentration of glycan was then determined using the same assay. This is given in Table S2. There are several additional sources of error: (i) the observed glycan-concentration is dependent on the ease of cleavage of the glycan from the CDs, which will be affected by the presentation of the glycan on the CD, which our AFM measurements demonstrate is inhomogeneous as discussed in the main manuscript; (ii) and a signal was also observed for the core-CDs however it is unclear whether this signal will remain for the glycan-CDs.

Table S2*. The relative glycan functionalisation for each CD species. Errors quoted are caused by the the 90% confidence intervals of fitting, the adjusted R-squared statistic for each of the carbohydrate calibration plots is also provided.*

CD species	Absorbance (490 nm)	Calibration factor (g / mM)	$\overline{R^2}$ of fitting	Glycan-functionalisation / CD mass (mmol g^{-1})
Core	0.123	-	-	-
Glucose	0.760	1.3±0.5	0.8954	0.6±0.2
Mannose	0.855	1.1±0.4	0.9314	0.8±0.3
Galactose	1.028	0.6±0.1	0.9760	1.7±0.3
Maltose	0.301	0.15±0.08	0.8038	2±1
Lactose	0.305	0.25±0.08	0.9253	1.2±0.4

Transmission Electron Microscopy (TEM)

TEM was performed on samples drop cast onto 4 nm thick carbon-coated 3mm-copper grids. The samples were prepared in HPLC grade H_2O at 5 mg/ml except for the lactose and core CDs which were prepared at 1 mg/ml and were dropped five times onto the same grid. The samples were all imaged on a Jeol 2100 TEM instrument at a voltage of 200kV. We were unable to attain sufficient contrast to directly image the glycan-functionalisation or the aromatic domains of the CDs via TEM measurements, due to the low electron density of surface carbohydrates as opposed to crystalline carbon. This is visualised in the imaging of the core as the crystalline lattice appears to span the particle, suggesting only the crystalline domain provides sufficient contrast (Figure S26). For functionalised CDs a slight increase in the radius, is however, still observed. The size distributions of each of the CDs are given in figures S20-S25. The size distributions agree with previous TEM measurements.[5] It should be noted that TEM is intrinsically an underestimate of particle size due to the low electron scattering cross section of the non-crystalline carbon regions, with previous studies reporting aromatic surface coverage.[5] For the core-CDs it is possible to image the crystalline lattice, (Figure S26). The lattice spacings observed by line profiles (Table S3) correspond to both sp^2 (100) and (020) or sp^3 (111) and (110), however for sp^2 carbon a lattice spacing of 0.33 nm corresponding to the (002) would also be expected. Such a conclusion is in agreement with our previous observations.[5] Example images of each of the glycan-CDs are shown in figures S27-S31.

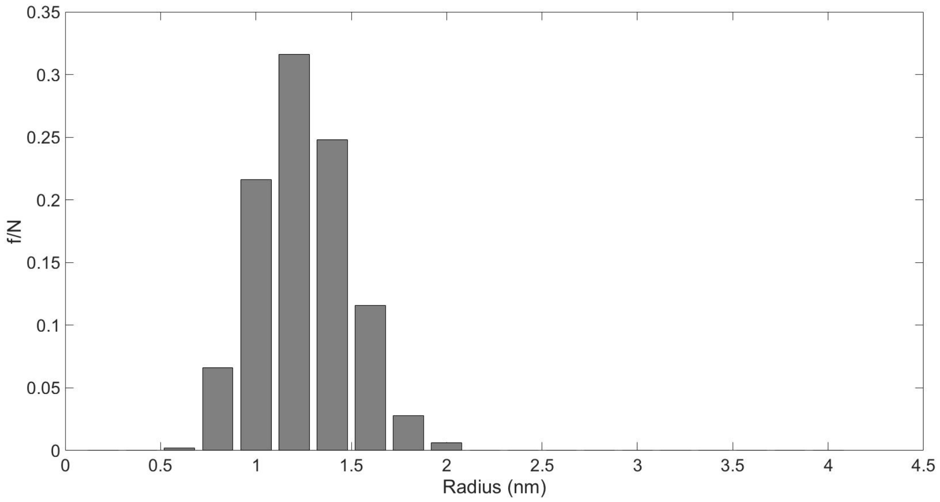

Figure S20: *Core-CD size distribution. N=500, the average radius = 1.25, standard error on the mean = 0.01nm, standard deviation = 0.2nm, median radius =1.23nm.*

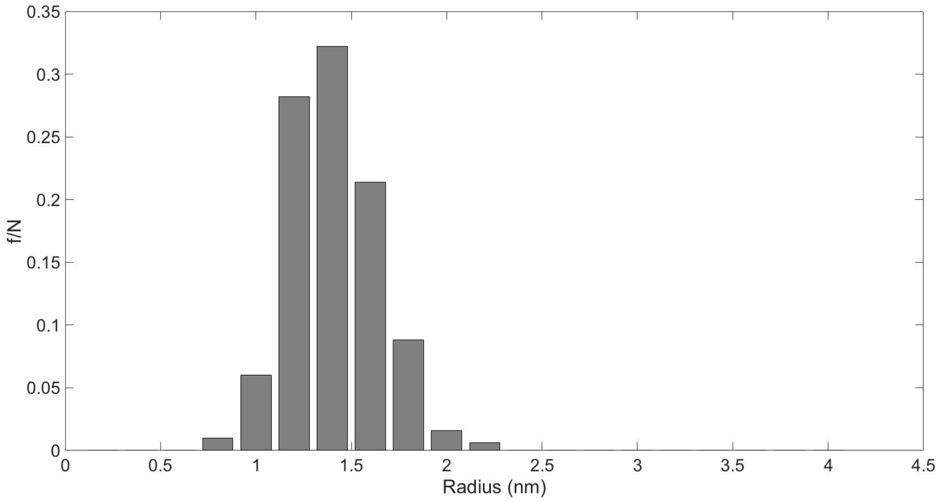

Figure S21: *Glucose-CD size distribution. N=500, the average radius = 1.41, standard error on the mean = 0.01nm, standard deviation = 0.2nm, median radius =1.38nm.*

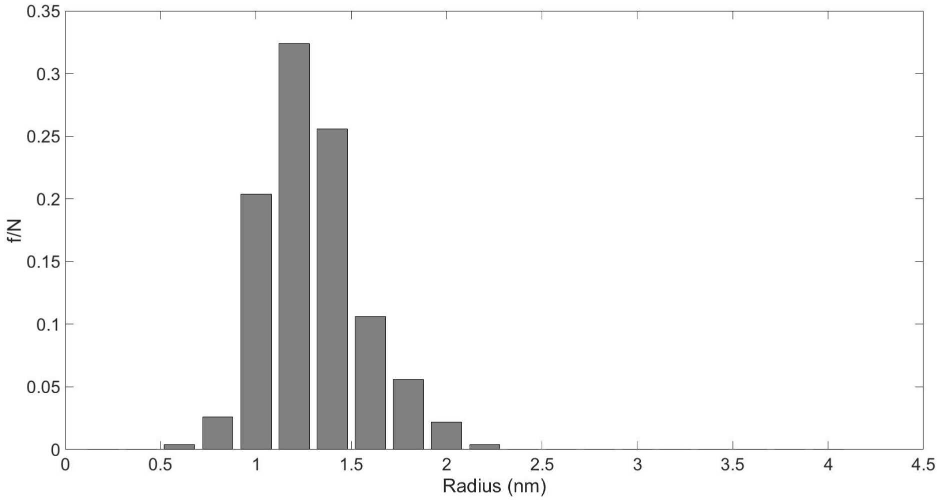

Figure S22: *Mannose-CD size distribution. N=500, the average radius = 1.294, standard error on the mean = 0.008nm, standard deviation = 0.2nm, median radius =1.256nm.*

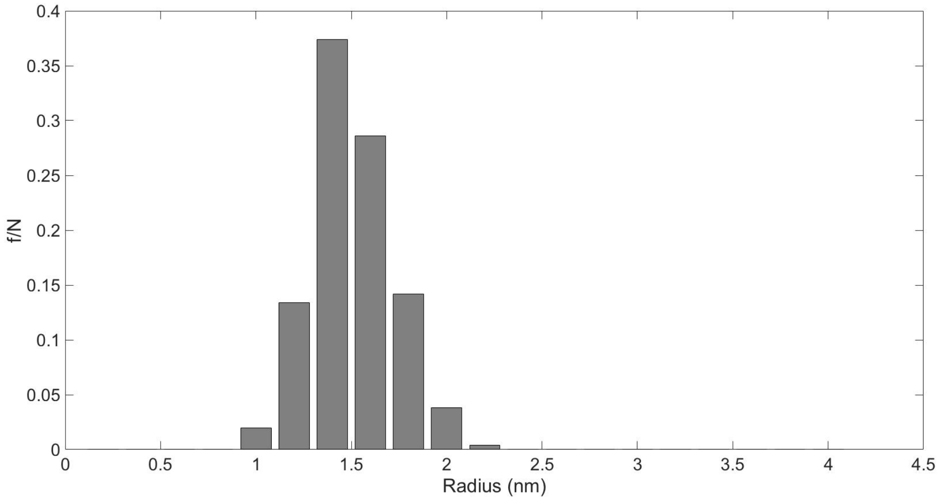

Figure S23: *Galactose-CD size distribution. N=500, the average radius = 1.512,*

standard error on the mean = 0.009nm, standard deviation = 0.2nm, median radius =1.489nm.

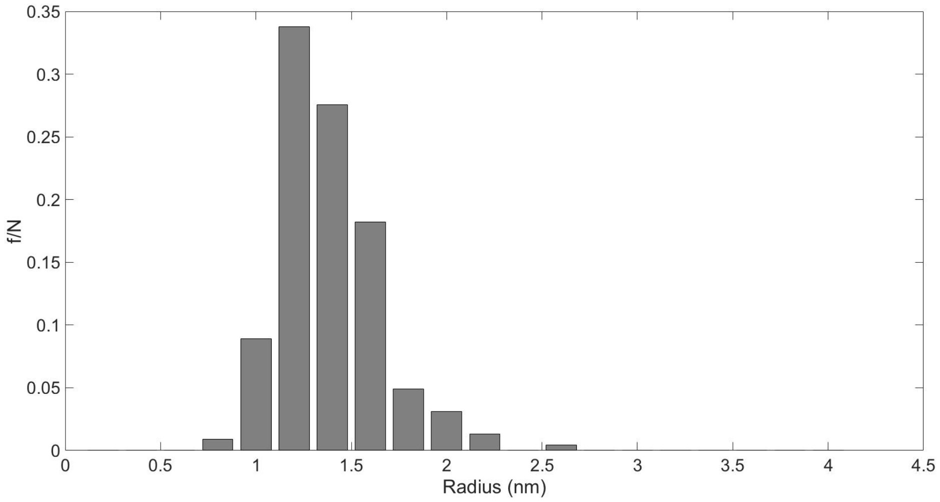

Figure S24: *Maltose-CD size distribution. N=225, the average radius = 1.45, standard error on the mean = 0.07nm, standard deviation = 1nm, median radius =1.33nm.*

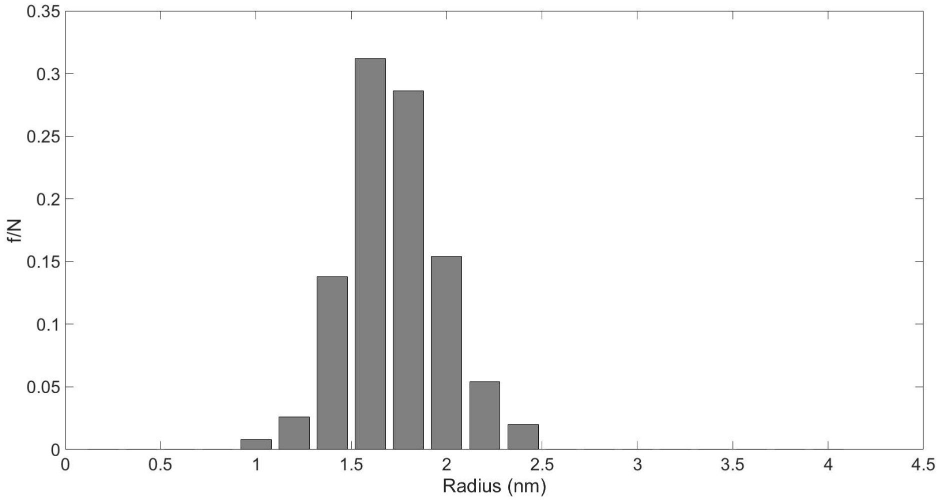

Figure S25: *Lactose-CD size distribution. N=500, the average radius = 1.72, standard error on the mean = 0.01nm, standard deviation = 0.2nm, median radius =1.71nm.*

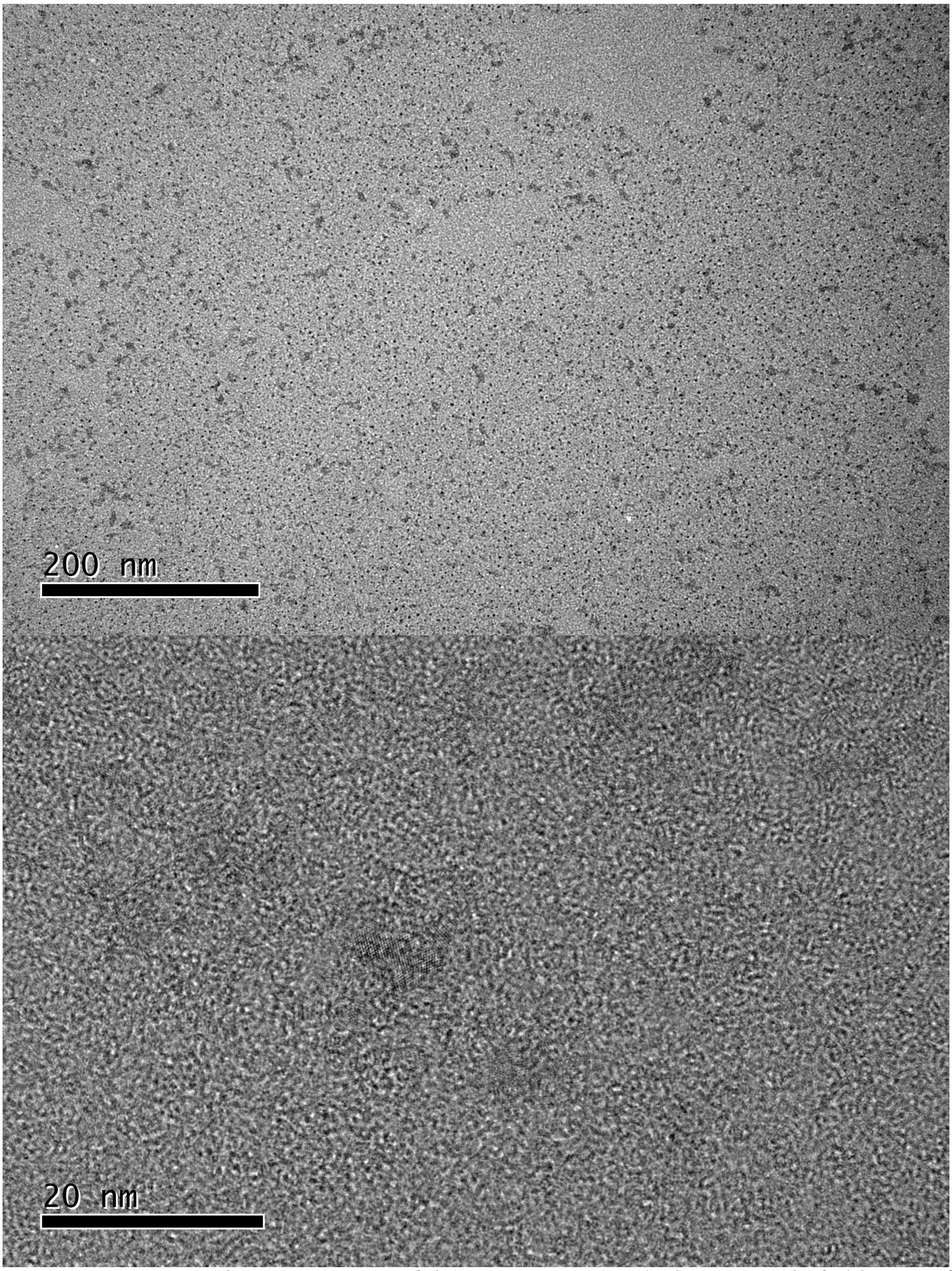

Figure S26: *Example TEM images of the Core-CDs: (Top) shows a wide field view of a population of dots; (bottom) shows several dots at high resolution to show the crystalline-carbon lattices. Lattice spacings were measured using line profiles (Table S3) Lattice spacings of 0.21 nm and 0.25 nm were extract from our TEM images correspond to the (111) and (110) of the* sp^3*-crystalline carbon, in agreement with our previous measurements.* [5] *It should be noted that the crystalline lattice spans the width of the particle, giving evidence that only the crystalline carbon is imaged with TEM.*

Table S3. *The lattice spacing calculated from line profiles from images of the core-CDs values are the average for each profile over the stated number of lattices, the error quoted is the error on the mean. Two different lattice spacings were observed, $\approx 0.25nm$ and $\approx 0.21nm$ which are shown on the right and left of the table respectively.*

Average lattice spacing (nm)	Number of lattices measured	Average lattice spacing (nm)	Number of lattices measured
0.25±0.01	10	0.20±0.01	6
0.26±0.01	9	0.202±0.008	12
0.26±0.01	9	0.19±0.01	8
0.26±0.02	10	0.21±0.01	9
0.28±0.02	10	0.21±0.01	10
0.26±0.02	10		
0.25±0.02	7		
0.24±0.02	8		

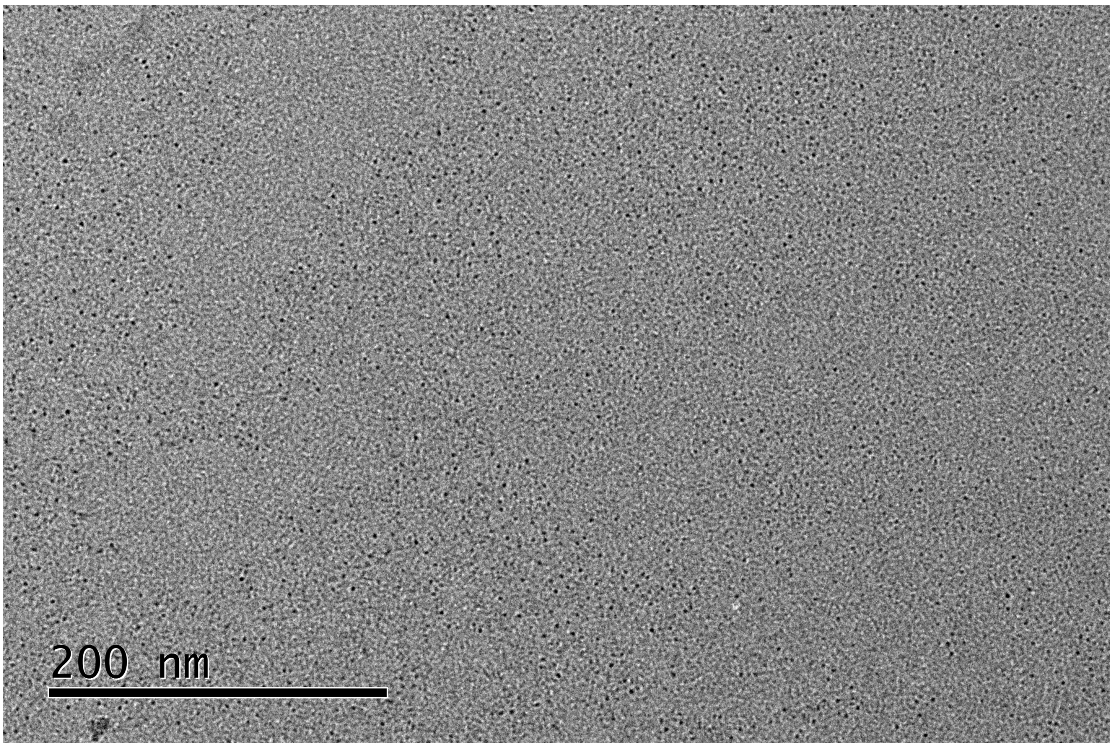

Figure S27: *An example TEM image of the Glucose-CDs.*

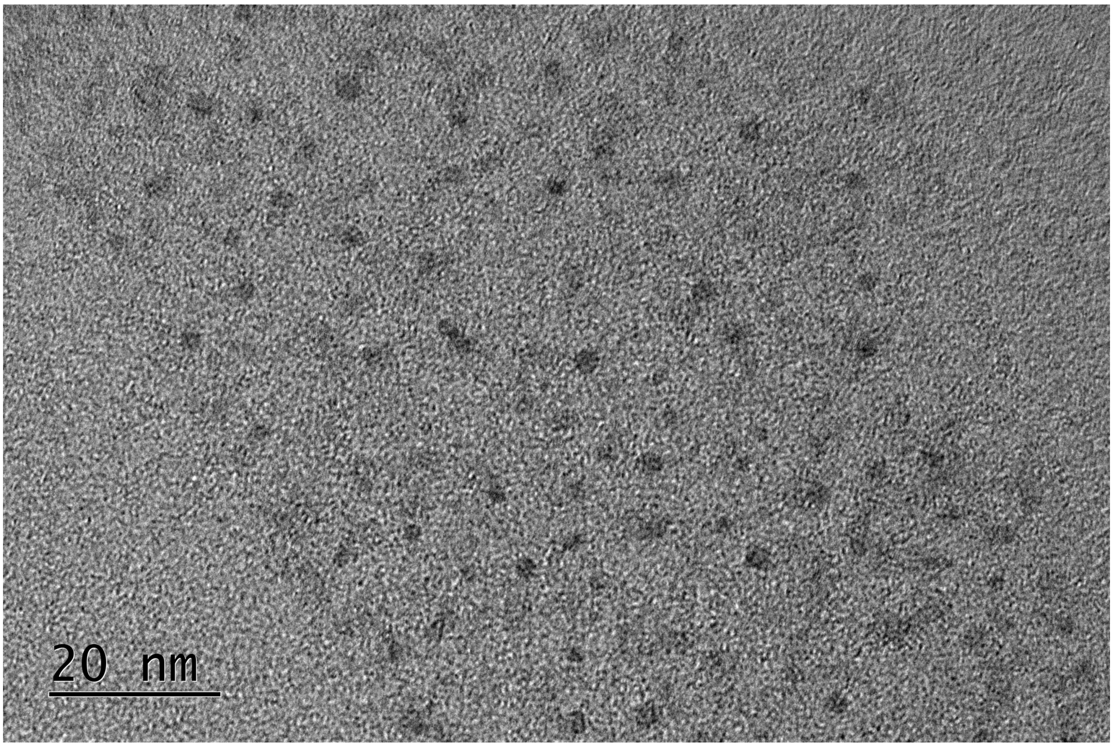

Figure S28: *An example TEM image of the Mannose-CDs.*

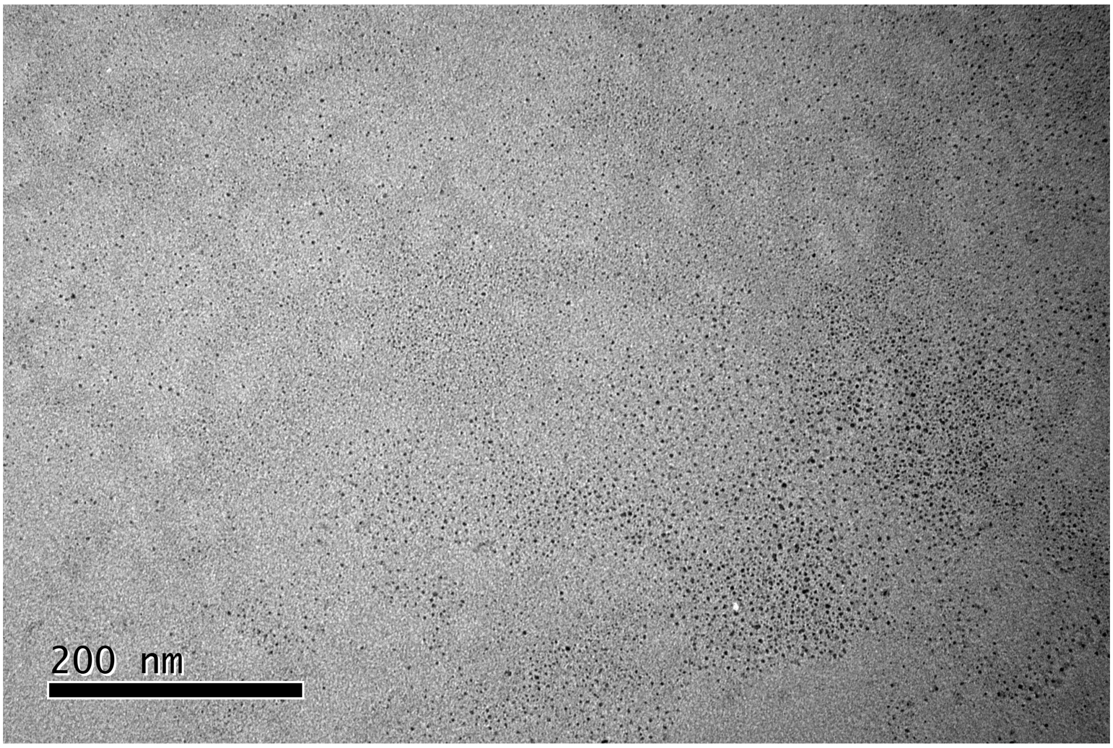

Figure S29: *An example TEM image of the Galactose-CDs.*

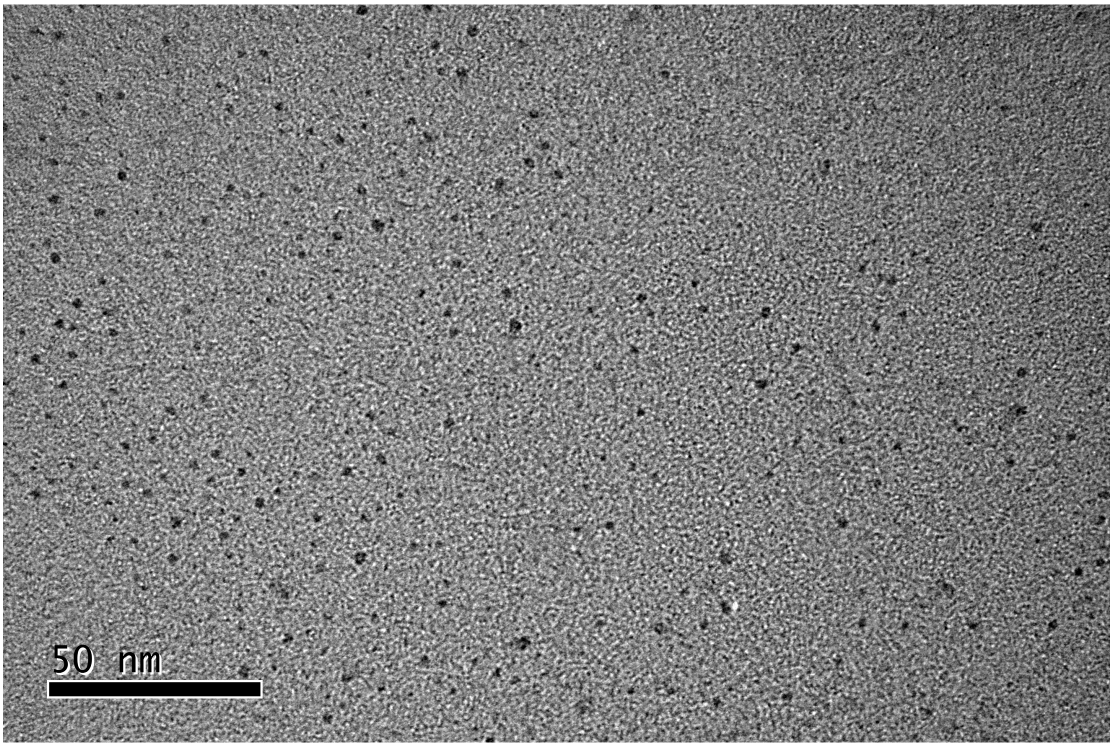

Figure S30: *An example TEM image of the Maltose-CDs.*

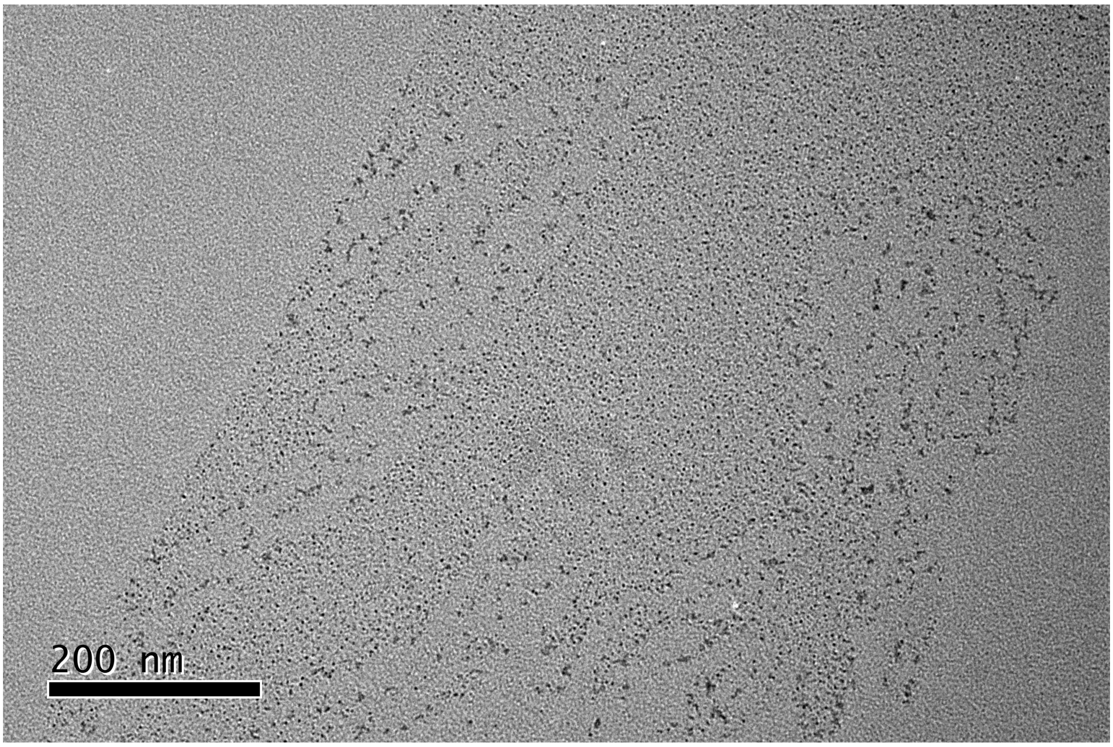

Figure S31: *An example TEM image of the Lactose-CDs.*

Small angle X-ray scattering (SAXS)

Small-Angle X-Ray scattering was conducted using a SAXSLAB Ganesha 300XL with a Pilatus 300K detector and an evacuated sample chamber. All samples were prepared in HPLC grade methanol and measured in 1.5mm borosilicate glass capillaries (Capillary Tube Supplies) sealed with UV-curable adhesive (Norland Optical Adhesive). Samples were run over a q range of 0.015-0.65 $Å^{-1}$. Mannose, lactose and maltose CDs had an exposure time of 60 minutes; the core, glucose and galactose samples had an exposure time of 30 minutes. Samples were background corrected with HPLC grade methanol. Initial data processing was carried out using SAXSGUI; Guiner analysis was undertaken using Matlab. From previous TEM studies, [5] the CD particles are known to have approximately 2 nm core; this was used to define the Guinier region. Guinier plots for each CD species are given in Figure S32. The raw data is shown in Figures S33.

Guinier analysis

The Guinier approximation was used to calculate the radius of the CD particles, and is found by the following approximation:

$$I_q = I_0 e^{\frac{-q^2 R_g{}^2}{3}} \qquad (2)$$

$$\log(I) = \ln(I_0) - \frac{q^2 R_g{}^2}{3} \qquad (3)$$

Where I is the intensity, R_g is the radius of gyration and q is the scattering vector is defined as:

$$q = \frac{4\pi \sin\theta}{\lambda} \qquad (4)$$

Where θ is the scattering angle λ is the wavelength of the source. The Guinier region is defined as when (with q in $Å^{-1}$ and R_g in Å):

$$q = \frac{1.3}{R_g} \qquad (5)$$

Remembering that for a sphere:

$$R_g{}^2 = \frac{3}{5} R^2 \qquad (6)$$

Where R is the radius. So for a sphere of predicted radius of ~2 nm, the range the Guinier approximation applies to the range:

$$q < 0.08Å^{-1}, q^2 < 0.007Å^{-2} \qquad (7)$$

The background methanol was subtracted and the data was used for the range 0.001 Å^{-2} < q^2 < 0.007 Å^{-2} , which returned the following results in figures S32. $\overline{R^2}$ is used to give the “adjusted r-squared” statistic.

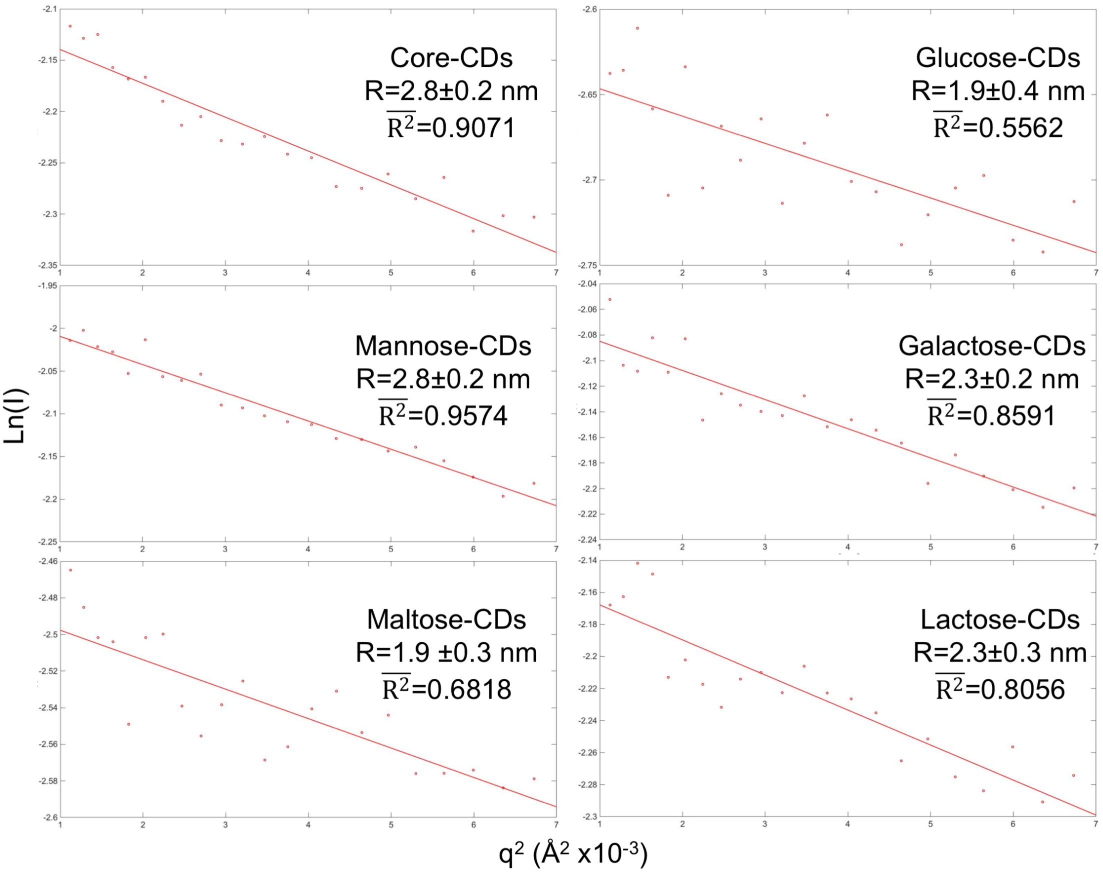

Figure S32: *Guinier plots for each of the CD species with their calculated radius (R) and the adjusted R-squared statistic for the fitting. Errors are quoted as the 90% confidence intervals.*

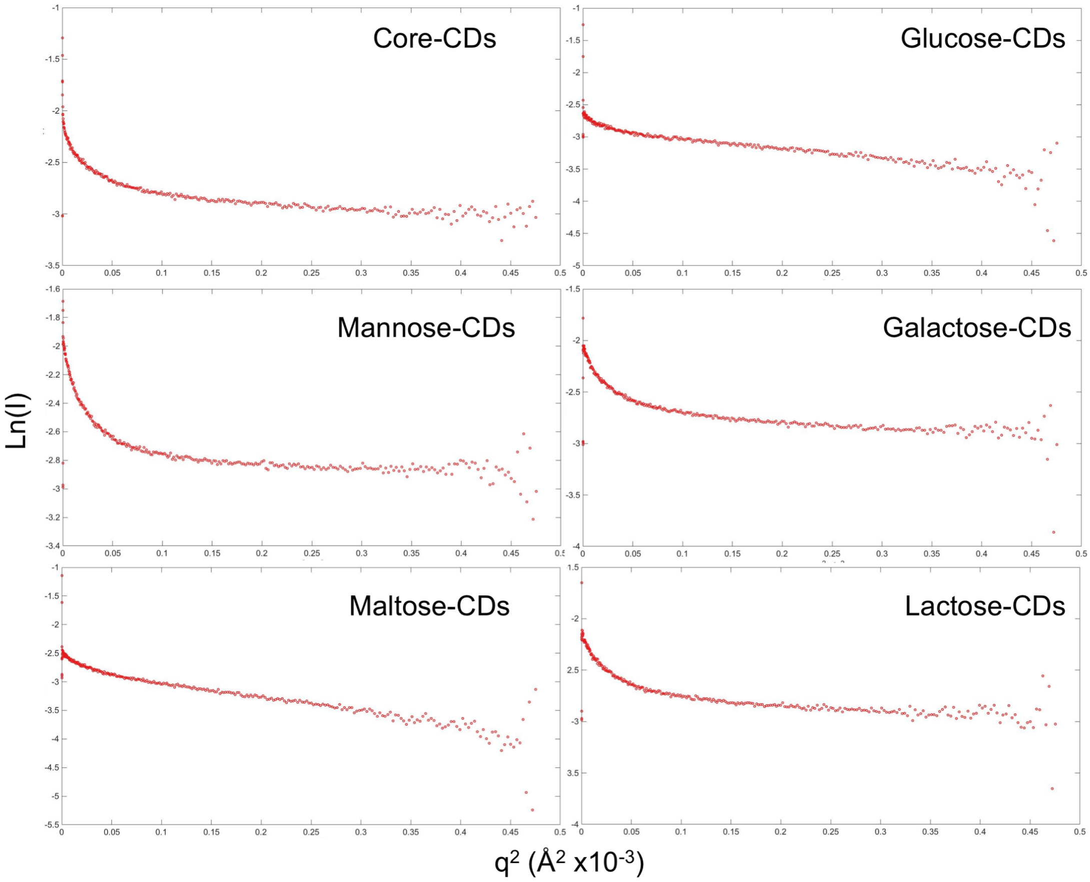

Figure S33: *The SAXS data for each of the CDs.*

Atomic Force Microscopy (AFM)

AFM measurements were collected utilising a Multimode VIII microscope with NanoscopeV controller operating with PeakForce feedback (Bruker, CA, USA). SCANASY ST-FLUID+ cantilevers were used with a nominal tip radius < 2 nm and spring constant of 0.7 Nm^{-1}. The samples were prepared by drop casting 15 μl of 0.1 $pgml^{-1}$ CDs in HPLC-grade methanol onto freshly cleaved mica; the excess methanol was blown off with nitrogen. The samples were allowed to dry for a minimum of 20 minutes before AFM experiments under ambient conditions.

For each CD sample, a single CD was imaged at high resolution to provide a detailed image of topography and adhesion. The tip-surface adhesion imaging gives further significant insights into the glycan-CD structure. For each glycan-functionalisation, several additional CDs have been imaged at lower resolution.

Tip-surface adhesion imaging gives further significant insights into the glycan-CD structure. These adhesion data reveal that the core is a low adhesion centre (dark blue), consistent with crystalline or aromatic carbon. The AFM measurements also show regions of high adhesion areas surrounding the core (light blue) associated with the glycan-corona, as expected for dense regions of the functionalised carbohydrates.

For the core CDs, we collected a large amount of data to investigate the typical core size distribution using AFM. The results are displayed as a histogram in Figure S34. Images of the core and glycan-functionalised CDs are given in figures S35-S39; the increase in adhesion for the glycan-CDs compared to the background is given in figure S40; a topographic map of the tip used to calculate the effective tip radius is given in Figure S42 and sample topographic profiles of each of the CDs are given in Figure S43.

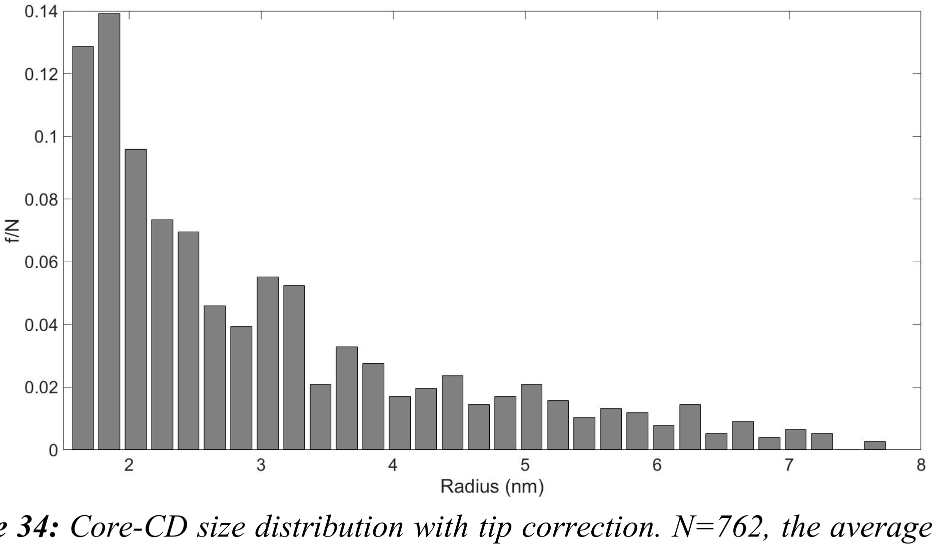

Figure 34: *Core-CD size distribution with tip correction. N=762, the average radius = 3.03, standard error on the mean = 0.05nm, standard deviation = 1nm, median radius =2.53nm. A topographic map of the tip is given in Figure S32. This data suggests a larger radius than previous TEM measurements. This is because TEM is most sensitive to crystalline regions, and may provide poor contrast for the surface functionalisations. Further, and for reasons given in the main manuscript AFM intrinsically over-estimates the size-distributions of particles sized <~ 2nm. It is also likely that the AFM includes some small aggregates where the sub-structure could not be resolved.*

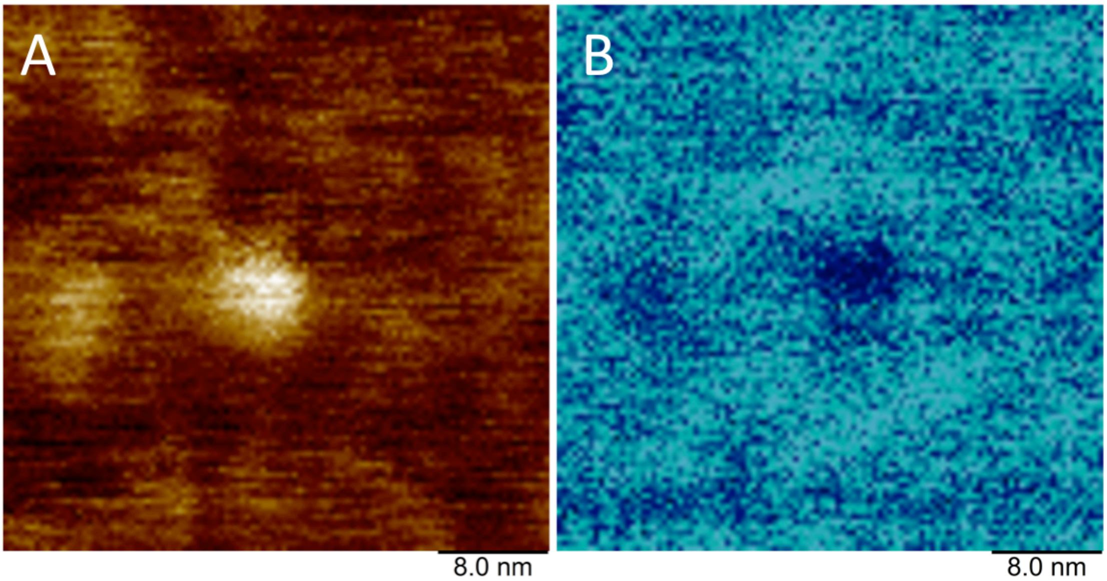

Figure S35: *Core-CD AFM: (A) high resolution image of topography, the height of the core is 1.13 nm and the radius is 3.34 nm, the height scale is 0-1.9 nm; (B) high resolution image of tip-surface adhesion interaction, the scale is 0-0.37nN, where regions of low adhesion are displayed in dark blue, and stronger adhesion in light blue.*

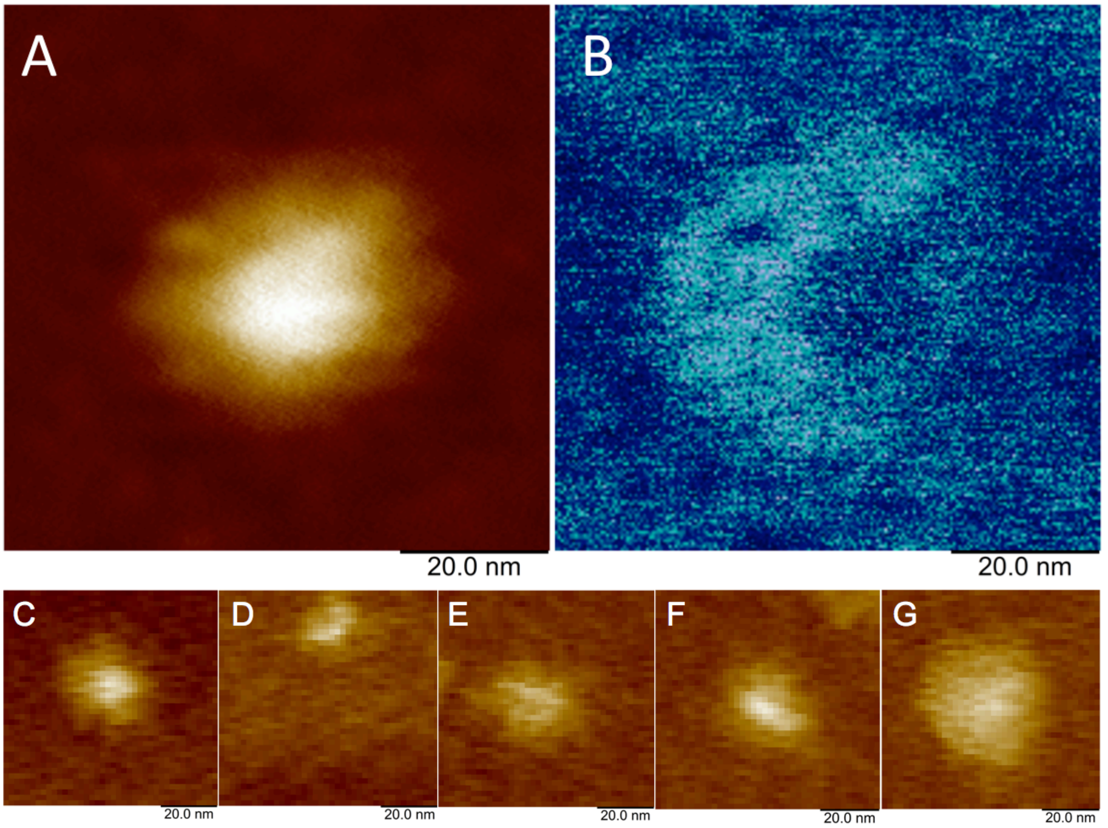

Figure S36: *Glucose-CD AFM: (A) high resolution image of topography, the height of the core is 3.55 nm and the radius is 5.69 nm, the functionalisation extends 15.42nm from the core, the height scale is 0-5.5 nm; (B) high resolution image of adhesion interaction, the scale is 0-0.49 nN; (C-G) topography images of additional Glucose-CDs, the height scale is 0-8nm.*

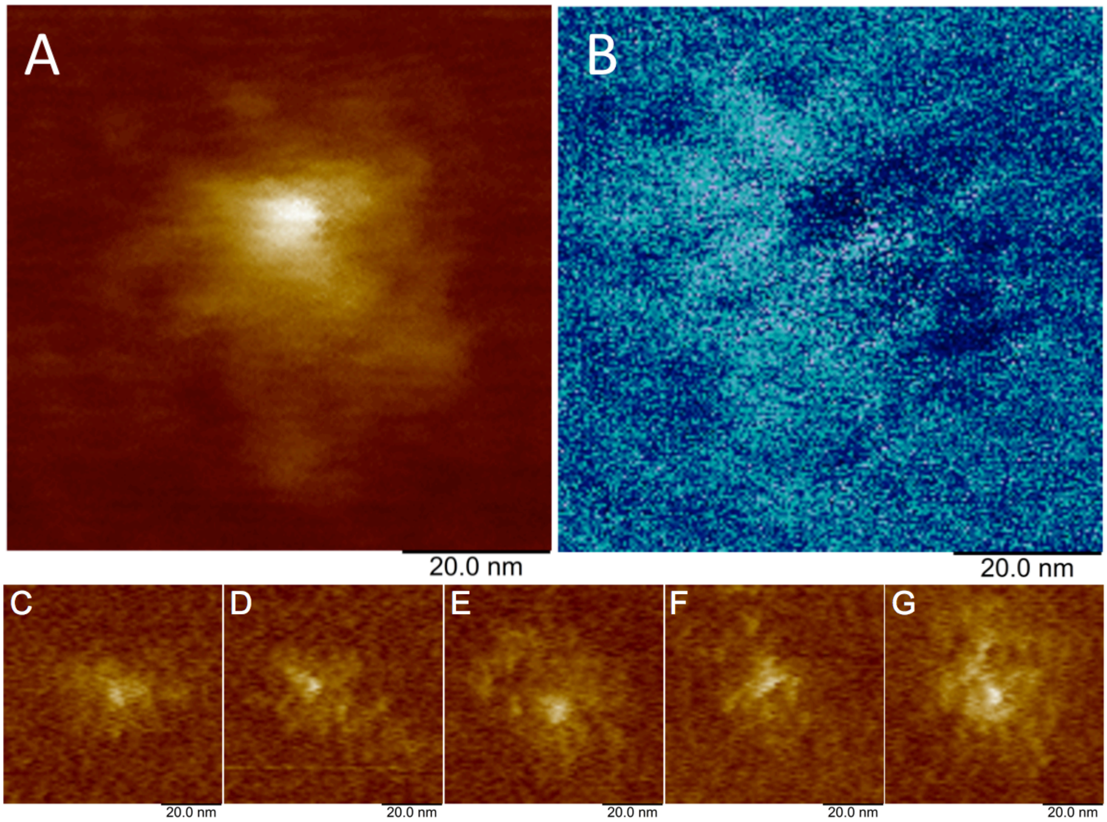

Figure S37: *Mannose-CD AFM: (A) high resolution image of topography, the height of the core is 3.38 nm and the radius is 3.92 nm, the functionalisation extends 16.11 nm from the core. The height scale is 0-5.8 nm; (B) high resolution image of adhesion interaction, the scale is 0-0.76 nN; (C-G) topography images of additional Mannose-CDs, the height scale is 0-6nm.*

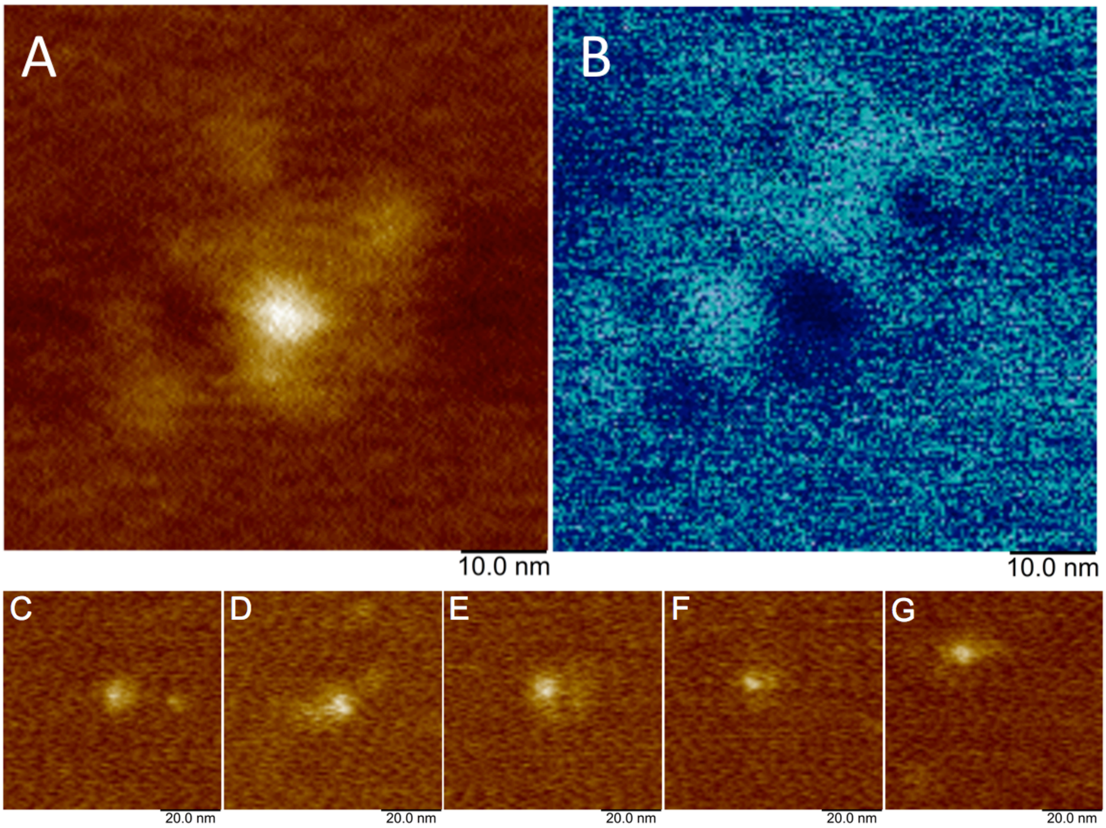

Figure S38: *Galactose-CD AFM: (A) high resolution image of topography, the height of the core is 1.21 nm and the radius is 2.94 nm, the functionalisation extends 12.15 nm from the core, the height scale is 0-2.2 nm; (B) high resolution image of the tip-surface adhesion interaction, the adhesion scale is 0-3.2 nN; (C-G) topography images of additional Galactose-CDs, the height scale is 0-6nm.*

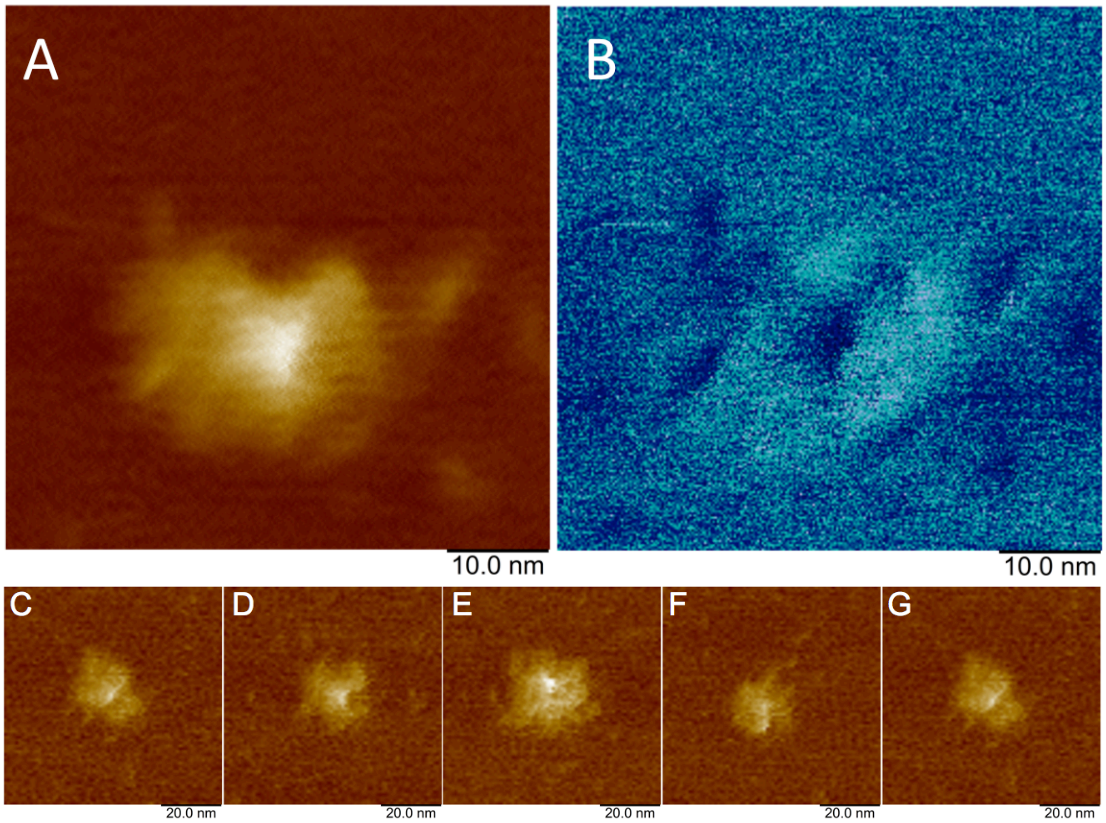

Figure S39: *Maltose-CD AFM: (A) high resolution image of topography, the height of the core is 2.27 nm and the radius is 2.70 nm, the functionalisation extends 10.92 nm from the core, the height scale is 0-4.5 nm; (B) high resolution image of tip-surface adhesion interaction, the adhesion scale is 0-0.11 nN; (C-G) topography images of additional Galactose-CDs, the height scale is 0-6nm.*

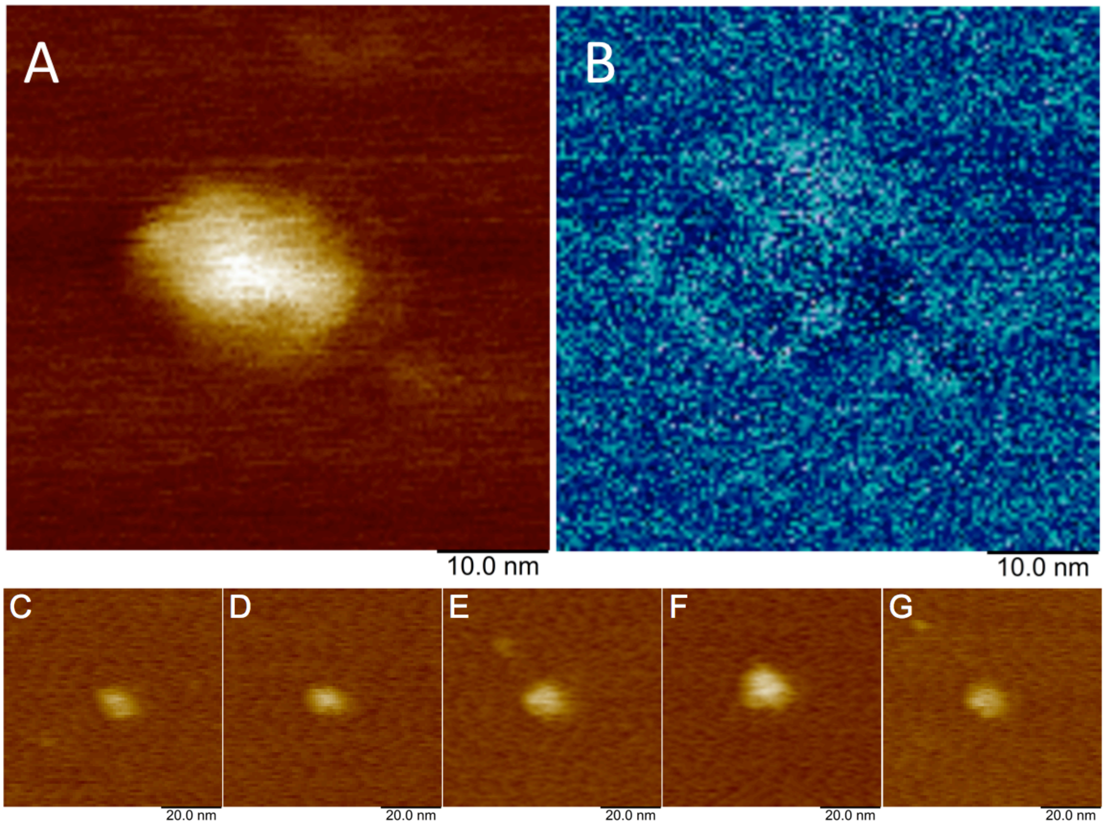

Figure S40: *Lactose-CD AFM: (A) high resolution image of topography, the height of the core is 2.18 nm and the radius is 2.94 nm, the functionalisation extends 7.74 nm from the core, the height scale is 0-3.5 nm; (B) high resolution image of the tip-surface adhesion interaction, the scale is 0-0.46 nN; (C-G) topography images of additional Galactose-CDs, the height scale is 0-6nm.*

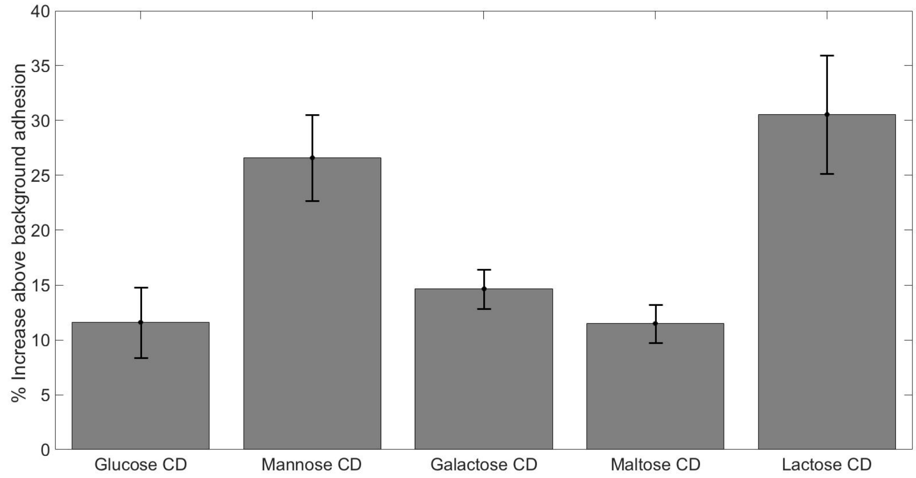

Figure S41: *Percentage increase of the range of tip-surface adhesion of the glycan-functionalised CDs normalised against the background mica. The adhesion of the mica is taken from equivalent sized surface areas. The error bars indicate the standard deviation. The adhesion of the CD is taken across the whole CD, the adhesion of the mica is taken from the rest of the same field. For each glycan functionalisation, between (14-35)×10^3 adhesion measurements are used.*

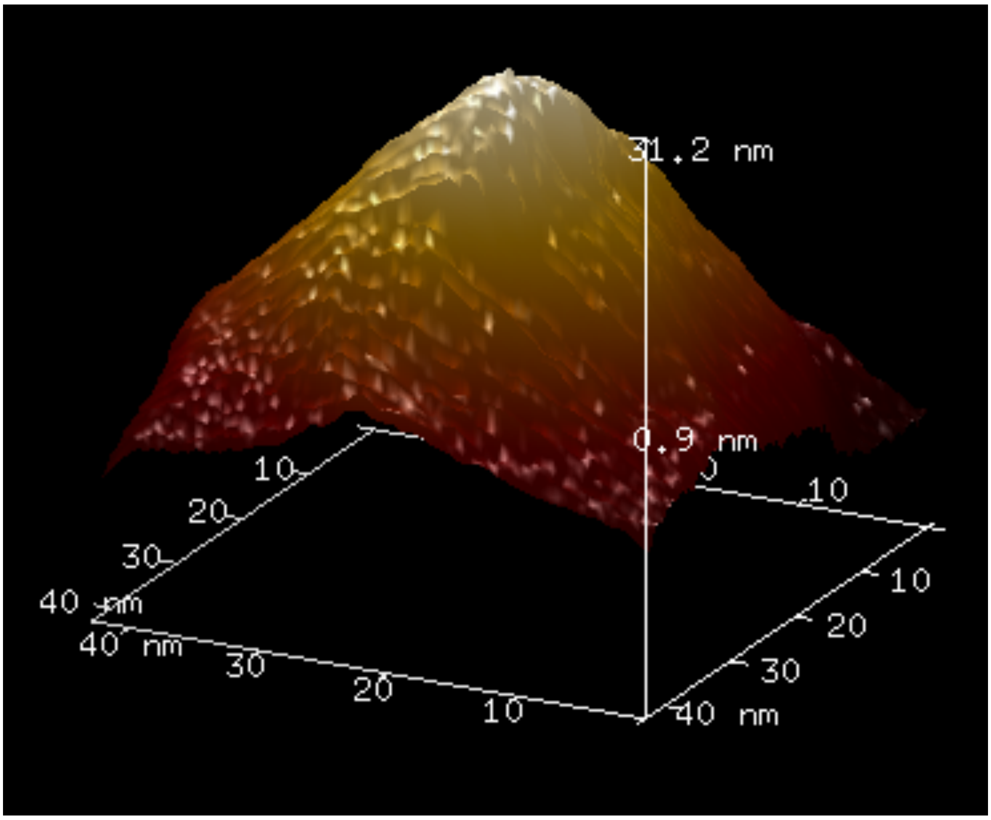

Figure S42: *A topography map of the AFM tip generated using tip-deconvolution on an aluminium grid. The effective tip radius was calculated to be approximately 0.35 nm.*

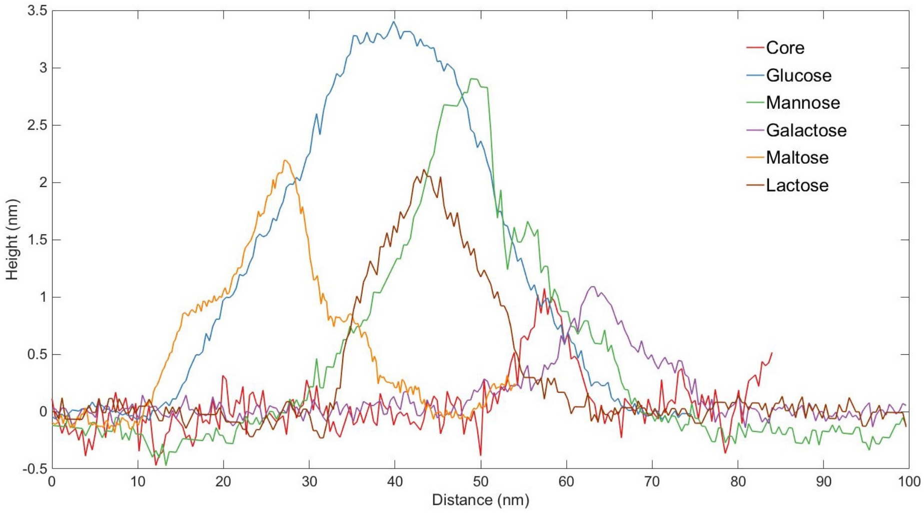

Figure S43: *Sample topographic profiles of the core-CDs and the glycan-functionalised CDs.*

Absorption Spectroscopy

The ultraviolet-visible absorption spectra of CDs were recorded using a Cary UV-Visible 50 spectrophotometer. The absorption spectra are dominated by bands at 273 and ~200 nm which are invariant to CD functionalisation. The absorption spectra at wavelengths longer than 315 nm are far more sensitive to glycan conjugation. This variation with functionalisation, which we also observe in the 2D fluorescence spectra, is ascribed to a modification of the electronic states which must be physically located on the surface of CDs. These most probably involve the aromatic shell that surrounds the crystalline sp^3 carbon core of the CDs.

TA experiments employed 340 nm pump excitation, and therefore excite a both core and surface states which have overlapping absorptions in this region.

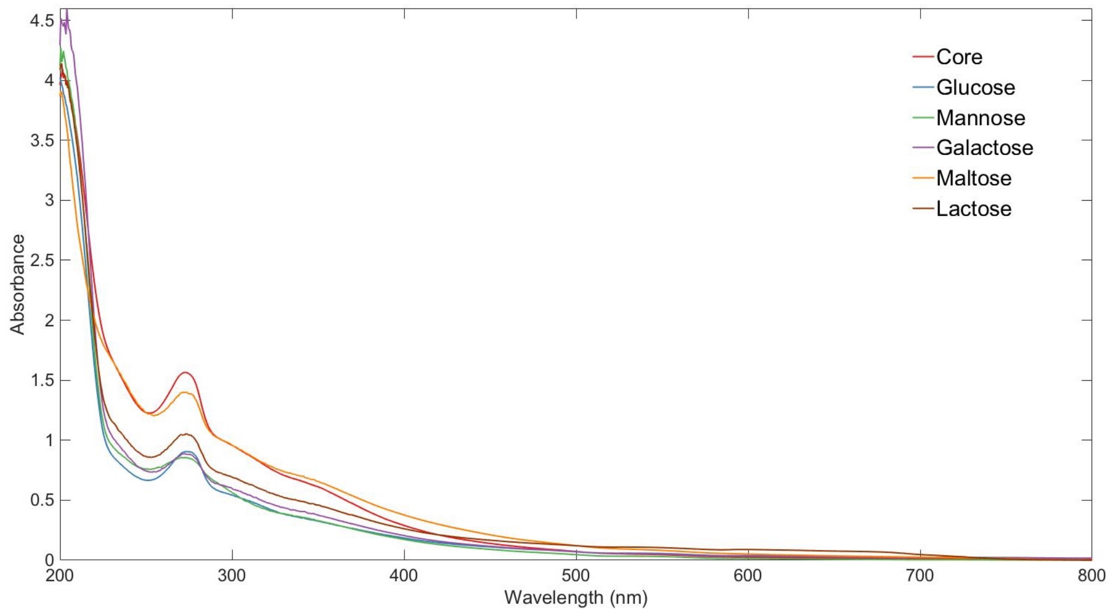

Figure S44: *UV-visible absorption spectra for the CDs in methanol between 200 and 800 nm. All of the spectra were taken at a concentration of 1.0 mg/ml in methanol in a 3mm path length quartz cell.*

Fluorescence Spectroscopy

Below are the fluorescence spectra for the CDs. All fluorescence measurements were recorded using a Perkin-Elmer LS45 spectrometer. All spectra were acquired for 1.0 mg/ml CD in methanol concentrations in a 3mm path length quartz cell.

The 2D fluorescence excitation-emission correlation spectra display at least one non-Kasha type fluorescence pathway at $\lambda_{emission}$ ~430 nm, which is blue-shifted relative to the main fluorescence band at ~ 465 nm. The intensity of the blue-shifted feature is minimal for core CDs. The intensity of this feature is far more prominent for functionalised CDs, but varies with the precise carbohydrate. This feature is correlated with the changes observed in the linear absorption spectra at λ > 315 nm, and from its position in the 2D fluorescence spectrum means excitation and emission are disconnected from the other absorption bands.

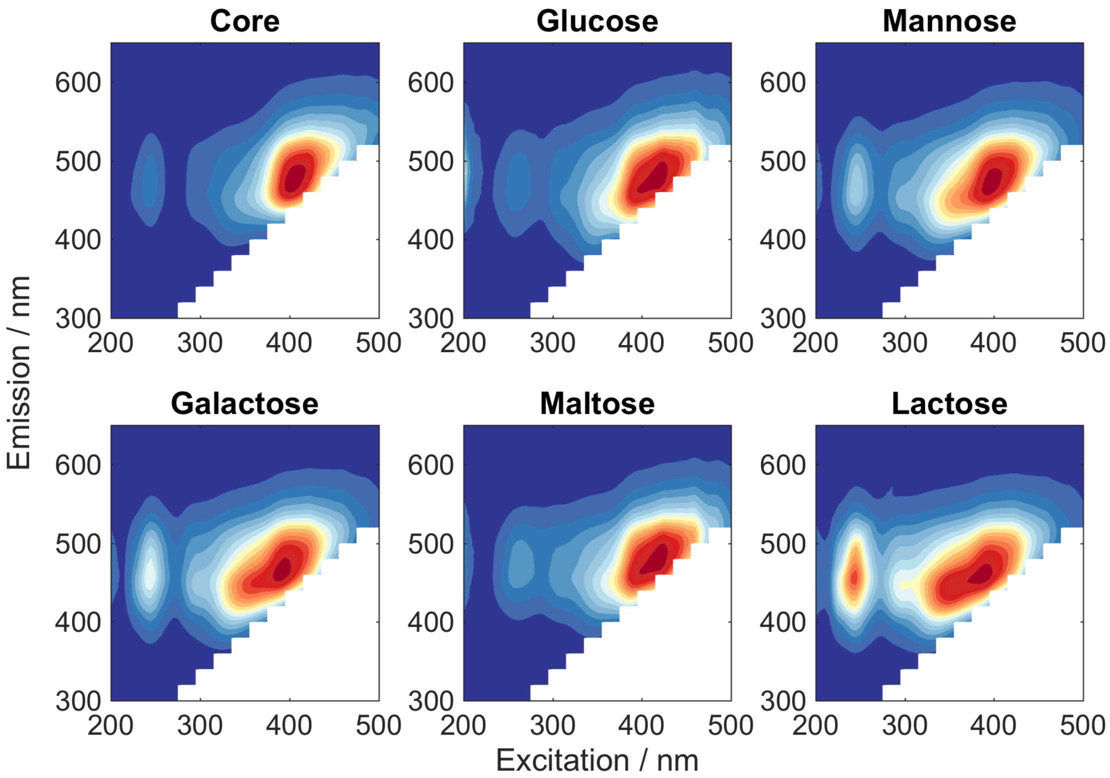

Figure S45: *Two-dimensional excitation-emission fluorescence spectra of CD samples.*

The quantum yield of the CDs was calculated using quinine sulphate as a reference. The data from the analysis is given in Figure S46 and the quantum yields tabulated in Table S4.

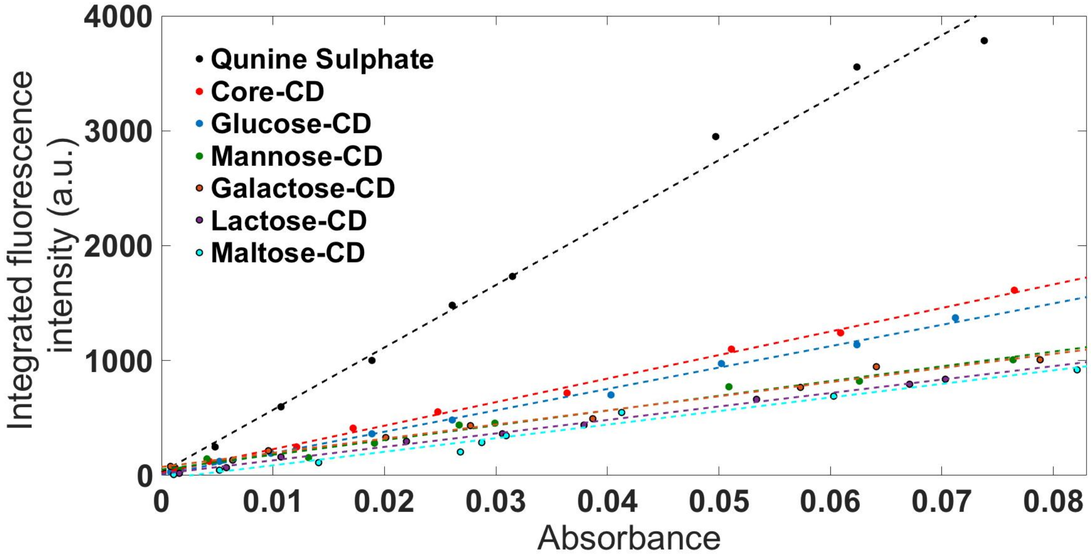

Figure S46: *Fluorescence quantum yield measurements for the CDs. The fluorescence was integrated across the emission spectrum at a 340nm excitation. All measurements were made in 0.1M H_2SO_4.Quinine sulphate was used as a control (known quantum yield =0.54) quantum yields are given in Table S4.*

Table S4. *Quantum yield values for each CD species. Errors in QY are the 90% confidence intervals of fitting.*

CD species	Quantum Yield	$\overline{R^2}$
Core	0.20±0.01	0.9964
Glucose	0.18±0.01	0.9956
Mannose	0.13±0.01	0.9801
Galactose	0.12±0.01	0.9838
Maltose	0.116±0.006	0.9960
Lactose	0.12±0.01	0.9739

Transient Absorption Spectroscopy

A Ti:Sapphire oscillator (Vitara-S, Coherent) was used to seed a regenerative amplifier (Legend Elite HE+, Coherent, ~40 fs, 800 nm) which was used to pump an optical parametric amplifier (OperA Solo, Coherent) generating 340 nm pump pulses (~100 fs). White light supercontinuum probe pulses (350–630 nm) were generated by focusing a very small portion of 800 nm light into a rastered CaF_2 plate (3 mm thick). At the sample position, the pump fluence was 600 nJ in a focal spot size of ~250 μm. The white light probe was focussed and overlapped with the 340 nm pump at the sample, with the transient absorption signals emitted collinearly to the probe beam. The probe and signal beams were collimated and imaged into a spectrograph (Shamrock 163, Andor) coupled to a 1024 element CCD array (Entwicklungsbüro Stresing). A copper sulfate solution was used to remove residual 800 nm from the white light supercontinuum before imaging into the spectrograph. The pump-probe time delay, t, was controlled with a mechanical delay stage (DDS220/M, Thorlabs). For all measurements the relative orientation of pump and probe pulses was set to magic angle. Data acquisition was performed in Labview. Transient absorption data were post-processed to compensate for the chirp in the white light probe using the Matlab suite. The global analysis was performed in Glotaran.

The concentration of carbon dots used in TA experiments was 1 $mgmL^{-1}$, corresponding to an absorbance of ~0.3 at 340 nm. CD samples were continuously flowed throughout TA experiments in a 1 mm path length Starna flow cell.

Transient Absorption Data

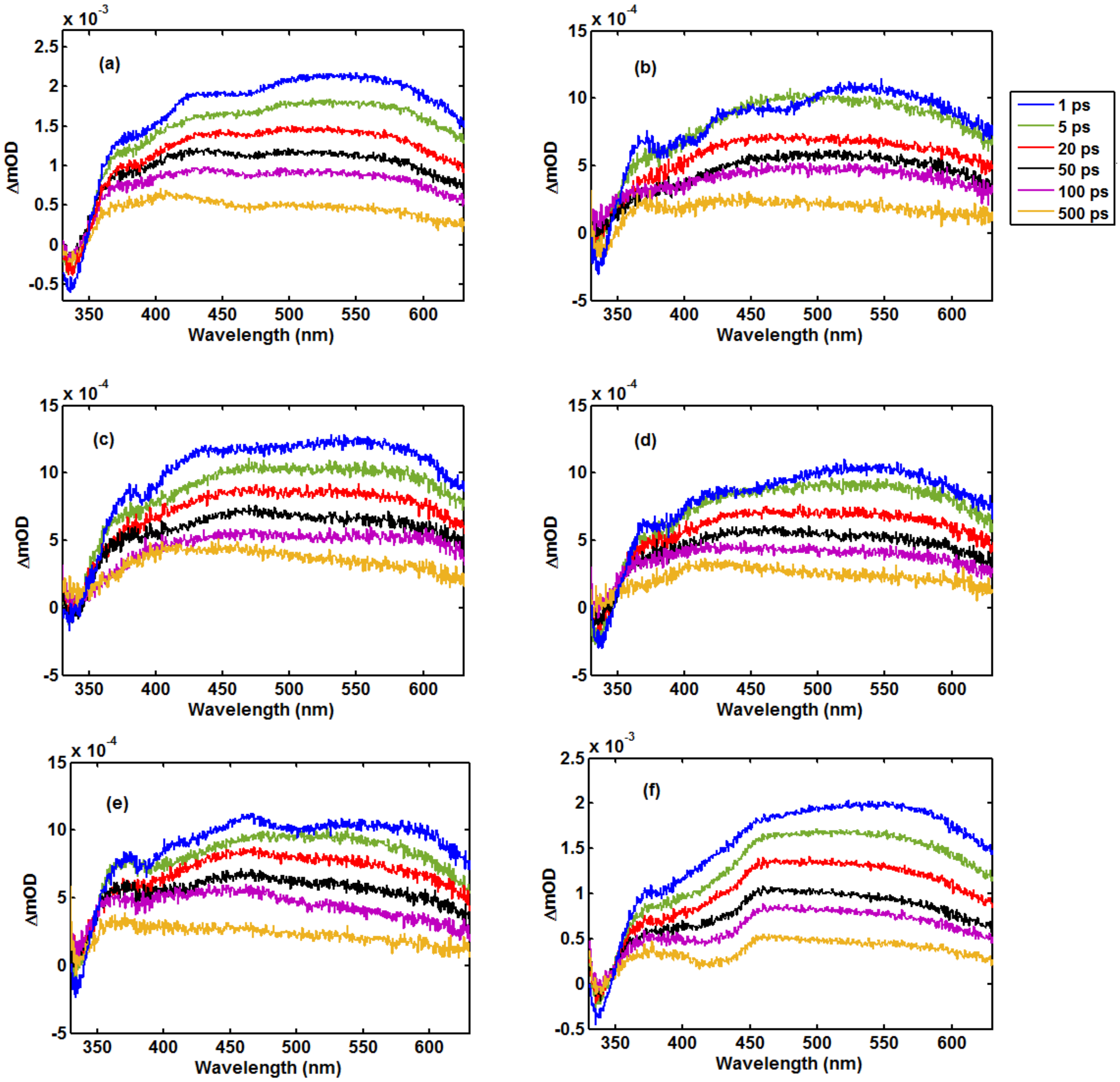

Figure S47: *Chirp-corrected TA spectra for (a) core, (b) galactose, (c) lactose, (d) maltose, (e) glucose (f) mannose samples.*

Global Analysis

A global analysis of the transient absorption data was performed in the Glotaran software package.[14] Such analyses avoid the problems associated with overlapping transient features and fits to data acquired at single wavelengths. The most reliable and physically plausible fits were returned by a sequential kinetic scheme (*i.e.* A → B → C) as opposed to a scheme that involved parallel decay pathways. These analyses decompose the transient absorption spectra into evolution assisted decay spectra (EADS) with associated concentration profiles. Unlike in a target analysis, the EADS merely reflect which spectral components decay that decay at the same rate, with an associated concentration profile. Each TA dataset for the various CD samples were modelled with three EADS components and returned very similar spectral profiles. Examples of fits to data in the frequency and time domains are given below for core CDs.

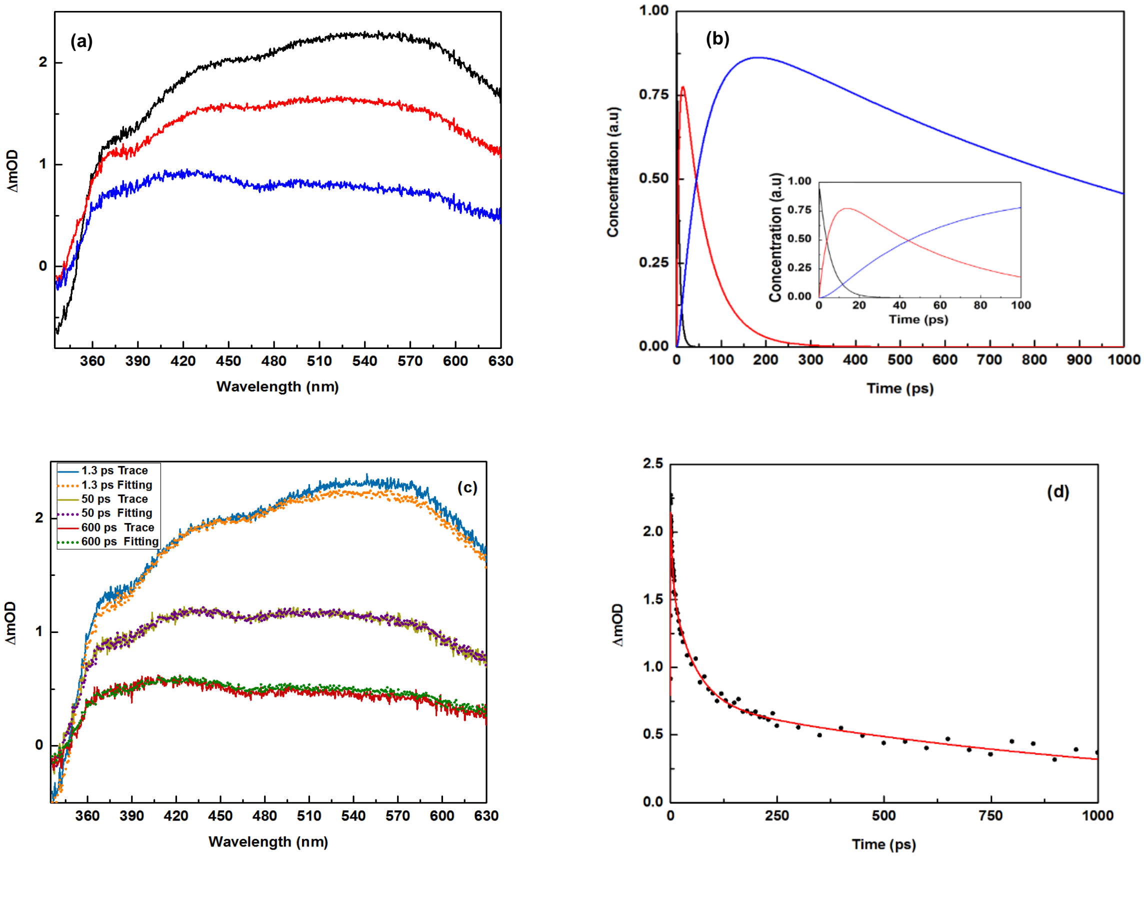

Figure S48: *Example fits returned from Global analysis for core dot TA data (a) three EADS, (b) color-coded concentration profiles associated with EADS spectra, inset displays concentration profiles for $0 \leq t \leq 100$ ps (c) Global analysis fits for t = 1.3, 50 and 600 ps (d) Raw data (dots) and global analysis (red line) fit to λ_{probe} = 580 nm.*

Zeta Potential

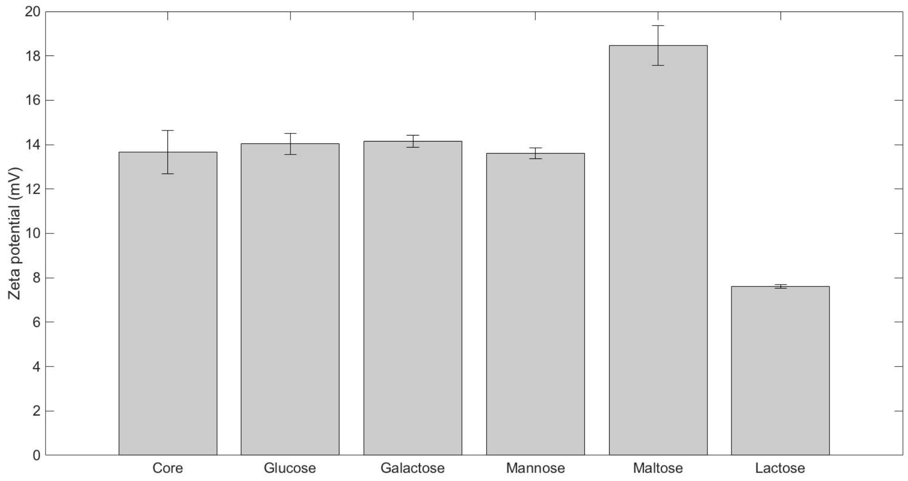

Figure S49: *Zeta potential for the different functionalisations in HPLC grade methanol. Each average is taken over 400 runs at 25℃ and at a concentration of 1mg/ml.*

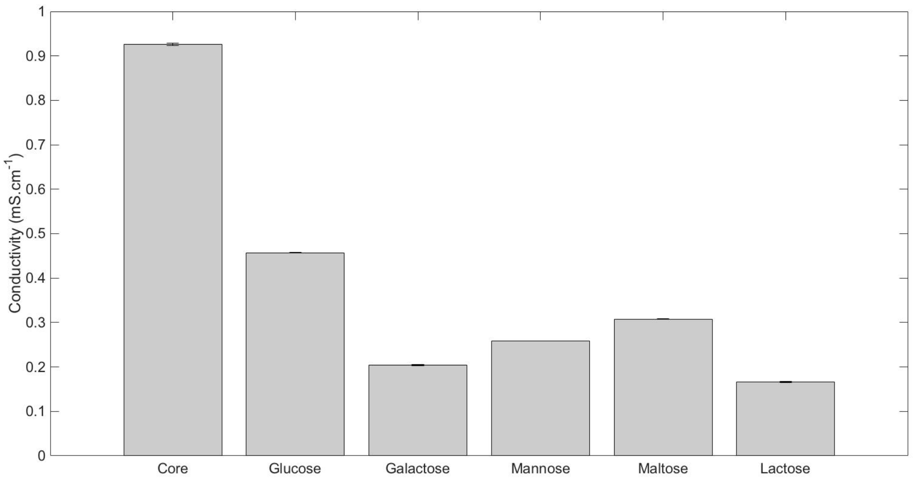

Figure S50: *Conductivity for the different functionalisations in HPLC grade methanol. Each average is taken over 400 runs at 25℃ and at a concentration of 1.0 mg/ml*

References

1 U. Dammer, O. Popescu, P. Wagner, D. Anselmetti, H. J. Güntherodt and G. N. Misevic, *Science*, 1995, **267**, 1173–1175.
2 W. I. Weis and K. Drickamer, *Annu. Rev. Biochem.*, 1996, **65**, 441–473.
3 Y. Ferrand, M. P. Crump and A. P. Davis, *Science*, 2007, **318**, 619–622.
4 K. L. Hudson, G. J. Bartlett, R. C. Diehl, J. Agirre, T. Gallagher, L. L. Kiessling and D. N. Woolfson, *J. Am. Chem. Soc.*, 2015, **137**, 15152–15160.
5 S. A. Hill, D. Benito-Alifonso, D. J. Morgan, S. A. Davis, M. Berry and M. C. Galan, *Nanoscale*, 2016, **8**, 18630–18634.
6 D. Benito-Alifonso, S. Tremel, B. Hou, H. Lockyear, J. Mantell, D. J. Fermin, P. Verkade, M. Berry and M. C. Galan, *Angew. Chem. Int. Ed.*, 2013, **53**, 810–814.
7 Mathias Nilsson, *J. Magn. Reson.*, 2009, **200**, 296–302.
8 L. Nagy, G. Gyetvai and G. Nagy, *Electroanalysis*, 2009, **21**, 542–549.
9 T. Yamanoi, Y. Oda and K. Katsuraya, *Magnetochemistry*, 2017, **3**, 38–6.
10 N. Mogi, E. Sugai, Y. Fuse and T. Funazukuri, *J. Chem. Eng. Data*, 2007, **52**, 40–43.
11 H. Ueadaira and H. Uedaira, *Bull. Chem. Soc. Jpn.*, 1969, **42**, 2140–2142.
12 A. C. F. Ribeiro, O. Ortona, S. M. N. Simões, C. I. A. V. Santos, P. M. R. A. Prazeres, A. J. M. Valente, V. M. M. Lobo and H. D. Burrows, *J. Chem. Eng. Data*, 2006, **51**, 1836–1840.
13 T. Masuko, A. Minami, N. Iwasaki, T. Majima, S.I. Nishimura and Y.C. Lee, *Analytical Biochemistry*, 2005, **339**, 69–72.
14 J. J. Snellenburg, S. P. Laptenok, R. Seger, K. M. Mullen and I. H. M. van Stokkum, *J. Stat. Software*, 2012, **49**, 1–22.
